# Orally Ingested Micro- and Nano-Plastics: A Hidden Driver of Inflammatory Bowel Disease and Colorectal Cancer

**DOI:** 10.3390/cancers16173079

**Published:** 2024-09-04

**Authors:** Annalisa Bruno, Melania Dovizio, Cristina Milillo, Eleonora Aruffo, Mirko Pesce, Marco Gatta, Piero Chiacchiaretta, Piero Di Carlo, Patrizia Ballerini

**Affiliations:** 1 Department of Innovative Technologies in Medicine & Dentistry, “G. d’Annunzio” University of Chieti-Pescara, 66100 Chieti, Italy; m.dovizio@unich.it (M.D.); cristina.milillo@unich.it (C.M.); eleonora.aruffo@unich.it (E.A.); marco.gatta@phd.unich.it (M.G.); p.chiacchiaretta@unich.it (P.C.); piero.dicarlo@unich.it (P.D.C.); patrizia.ballerini@unich.it (P.B.); 2 Center for Advanced Studies and Technology (CAST), “G. d’Annunzio” University of Chieti-Pescara, 66100 Chieti, Italy; 3 Department of Medicine and Aging Sciences, “G. d’Annunzio” University of Chieti-Pescara, 66100 Chieti, Italy; mirkopesce@unich.it; 4 UdA—TechLab, Research Center, “G. d’Annunzio” University of Chieti-Pescara, 66110 Chieti, Italy

**Keywords:** microplastics, nanoplastics, human exposure, toxicity, inflammatory bowel diseases, intestinal tumorigenesis

## Abstract

**Simple Summary:**

The increasing production of plastics and the failure to properly manage their post use represent the primary sources of environmental and human contamination caused by plastic degradation products, namely micro- and nano-plastics (MNPLs). Accumulating evidence supports the intestinal toxicity of ingested MNPLs, and their possible role as a driver for the increased incidence of colorectal cancer (CRC) has also been proposed. Moreover, colonic mucus layer disruption may further facilitate MNPL passage into the bloodstream, thus promoting the toxic effects of MNPLs on different organ systems. We herein discuss the pathophysiological mechanisms impaired by MNPL oral exposure in healthy conditions and individuals with compromised intestinal barrier function, including inflammatory bowel disease (IBD) and CRC patients, considered high-risk populations, by highlighting the potential contribution of MNPL-induced platelet activation.

**Abstract:**

Micro- and nano-plastics (MNPLs) can move along the food chain to higher-level organisms including humans. Three significant routes for MNPLs have been reported: ingestion, inhalation, and dermal contact. Accumulating evidence supports the intestinal toxicity of ingested MNPLs and their role as drivers for increased incidence of colorectal cancer (CRC) in high-risk populations such as inflammatory bowel disease (IBD) patients. However, the mechanisms are largely unknown. In this review, by using the leading scientific publication databases (Web of Science, Google Scholar, Scopus, PubMed, and ScienceDirect), we explored the possible effects and related mechanisms of MNPL exposure on the gut epithelium in healthy conditions and IBD patients. The summarized evidence supports the idea that oral MNPL exposure may contribute to intestinal epithelial damage, thus promoting and sustaining the chronic development of intestinal inflammation, mainly in high-risk populations such as IBD patients. Colonic mucus layer disruption may further facilitate MNPL passage into the bloodstream, thus contributing to the toxic effects of MNPLs on different organ systems and platelet activation, which may, in turn, contribute to the chronic development of inflammation and CRC development. Further exploration of this threat to human health is warranted to reduce potential adverse effects and CRC risk.

## 1. Introduction

A constant increase in plastic production is set to persist until 2050 [1]. Plastics can contaminate the environment due to ocean currents, winds, and atmospheric phenomena, promoting their distribution [2]. Once released, plastic degradation occurs, leading to the formation of microplastics (MPs) (smaller than 5 mm) and nanoplastics (NPs) (smaller than 1000 nm) [3,4]. It has been well recognized that micro- and nanoplastics (MNPLs) can penetrate the human body by ingestion, inhalation, and skin exposure [4,5]. They have been detected in some human tissues and biological samples, including the placenta [6], lungs [7], liver [8], breast milk [9], urine [10], and blood [11]. A large body of evidence suggests that MNPL exposure may cause a wide spectrum of adverse effects in human organ systems, including oxidative stress, inflammation, alteration in immune function and cellular and energy metabolism, suppression of cell proliferation, tissue damage and degeneration, organ development and function, biochemical parameters, and genotoxic and carcinogenic effects [12]. In addition, MNPLs bear a high toxicological risk because they incorporate harmful chemical additives including plasticizers, flame retardants, stabilizers, dyes, antistatic agents, lubricants, flow agents, foaming agents, and biocides [13]. MNPLs may adopt a fibrous shape, commonly termed “microplastic fibers” [14]. The environmental contamination by MNPL fibers is comparable to or even higher than that caused by plastic particles [15,16]. The elongated shape of microplastic fibers potentially increases their bioaccumulation and may directly damage organisms or cause adverse effects.

Worldwide, about 39,000–52,000 particles per person are ingested every year by food consumption [17]. The intestine is considered the primary line of defense against orally ingested MNPLs. Once ingested, they may impair intestinal barrier structures, alter intestinal flora, and induce mild pro-inflammatory responses and oxidative stress in healthy individuals [18,19,20,21]. However, the extent of damage to the intestine is not considered enough to determine an intestinal disease state [22,23], thus suggesting the capacity of a healthy intestine to resist the impact of MNPLs [24]. On the other hand, there is a great deal of evidence supporting the idea that MNPLs could affect the severity of diseases such as obesity, alcoholic liver injury, and enteritis [25,26,27]. Moreover, the integrity of the intestinal barrier is closely associated with developing diseases including neurodegenerative disease and inflammatory bowel disease (IBD) [28,29,30].

IBD incidence generally increases in developing industrialized countries due to severe environmental pollution [31,32,33]. In IBD patients, the intestinal microenvironment is different from that of healthy individuals, and MNPLs could significantly increase intestinal injury and exacerbate intestinal pathology development [34,35,36]. However, knowledge about the impact of MNPLs on the IBD population and related mechanisms is still limited.

Recently, the finding that the number of MNPLs in human colorectal cancer (CRC) biopsies is significantly higher than those detected in non-tumoral colon biopsies has suggested the occurrence of a connection between CRC development and MNPL exposure level [37]. This possible link is sustained by the increased CRC incidence in the under-50 population [38,39]. This recent epidemiological data may reflect the period potentially expected to see the effects associated with the fast increase of MNPLs in the environment.

Intestinal damage and inflammation caused by ingested MNPLs contribute to their entering the bloodstream and disseminating to other tissues. For example, once they reach systemic circulation, MNPLs may represent a risk to the cardiovascular system. Though knowledge about the cardiovascular effects of MNPL exposures is still limited, the presence of MNPLs in multiple human heart tissues and blood has recently been evidenced [40]. Overall, MNPLs may cause vascular endothelial damage, promoting a series of adverse cardiovascular effects via the interaction between endothelial cells and blood and immune cells [12]. In vivo studies in animal models support the capacity of MNPLs to alter heart rate and cardiac function to promote inflammatory responses, myocardial fibrosis, and endothelial dysfunction [41]. Experimental evidence also supports the idea that cardiovascular toxic effects by MNPLs may be associated with their capacity to affect platelet aggregation and blood coagulation. MNPLs may exert prothrombotic effects depending on different factors, including their surface modification, size, dose, exposure route, and protein presence. MNPL exposure may have an impact on the balance between pro- and anticoagulant pathways at molecular levels and affect blood coagulation through different mechanisms, mainly depending on particle size and surface modification [41]. However, the clinical relevance of these findings is largely unexplored. Recently, Marfella et al. [42] showed that patients with carotid artery plaque, in which MNPLs were detected, had an increased risk of a composite of myocardial infarction, stroke, or death (at 34-month follow-up) compared to patients in whom MNPLs were not evidenced. However, in the last decades, during which plastic exposure has presumably increased, the occurrence rate of cardiovascular disease has decreased [43]. This suggests that, if compared with common risk factors, the potential role of MNPLs in triggering cardiovascular disease might be limited. Nevertheless, the impact of MNPL exposure in this setting deserves further investigation.

Several lines of evidence support the contribution of platelets to the chronic development of intestinal inflammation. Moreover, it has been shown that the chronic use of the antiplatelet agent low-dose aspirin for cardiovascular prevention reduces CRC incidence and mortality [44]. Altogether, these findings support the hypothesis that activated platelets can trigger chronic inflammation, representing a key event in intestinal tumorigenesis [44]. Once activated in response to intestinal epithelial damage, platelets contribute to acute inflammation by promoting leukocyte recruitment. This is essential to restore normal tissue function. However, uncontrolled platelet activation leads to the increased release of lipid mediators including thromboxane (TX)A_2_ and the prostaglandin (PG)E_2_ as well as growth factors, angiogenic factors, cytokines, chemokines, and medium-sized extracellular vesicles (EVs) containing genetic material (such as mRNA and microRNAs). These platelet-derived mediators promote cyclooxygenase (COX)-2 expression and PGE_2_ biosynthesis by tumor microenvironment cells (i.e., immune and endothelial cells and fibroblasts, as well as cancer cells). These events are considered key points for chronic development of inflammation, tumor progression, angiogenesis, and metastasis [44].

The omnipresence of plastics in our daily lives implies chronic exposure to MNPLs. However, there are still numerous knowledge gaps about the harmful effects of MNPL exposure on human health. Ethical considerations hamper clinical studies investigating the health risks of MNPL exposure in humans. Moreover, the lack of data on plastic levels in the human diet does not allow a robust scientific conclusion for humans in general and their intestinal health. It is clear that plastics contaminate water and the food chain, and a growing body of experimental evidence from in vitro and animal studies shows that MNPL ingestion disrupts essential intestinal functions such as the gut barrier function and regulation of the gut microbiota [45]. These plastic-associated effects may promote immune, inflammatory, and metabolic disorders and therefore warrant further investigation. Recently, epidemiological data suggested that CRC incidence has been increasing worldwide in individuals under 50 years of age, thus supporting the occurrence of a cohort effect since 1960. The etiology of this observation is unknown, but it is likely promoted by an environmental cause [46]. In this review, we provided a comprehensive overview of current knowledge about the potential effects of ingested MNPLs on the intestinal tract in healthy individuals. We also explored possible mechanisms of how MNPL exposure may exacerbate the chronic development of inflammation and promote intestinal tumorigenesis in IBD patients, highlighting the potential contribution of MNPL-induced platelet activation.

With this aim, the leading scientific publication databases (Web of Science, Google Scholar, Scopus, PubMed, and ScienceDirect) were used to collect and select relevant literature for clearly focused questions about the impact of MNPL on human health. They include: “microplastics”, “nanoplastics”, “detection techniques”, “oral ingestion”, “gastrointestinal tract”, “intestinal health”, “inflammatory bowel disease”, “colorectal cancer”, “animal models”, “in vitro studies”, “humans”, “oxidative stress”, “apoptosis”, and “platelets”. Furthermore, to highlight other pertinent literature in the publications we obtained, we cross-checked the sources cited. Articles were sorted by title and abstract; only full publication data were analyzed. Conferences, books, encyclopedias, editorials, letters, and non-English articles were excluded. The analysis was performed separately, one by one, and the data were visualized accordingly. The latest literature search was conducted in August 2024, and priority was given to articles published in high-impact journals during the last ten years.

## 2. Micro- and Nanoplastics (MNPLs): An Overview

### 2.1. Definition and Composition of MNPLs

Although the first use of plastics can be traced back to the 2nd millennium BC, the invention of modern natural plastic is attributed to Charles Goodyear, who patented the vulcanization process of natural rubber in 1844. Later, synthetic plastic (bakelite) was invented by Leo Baekeland in 1907. Subsequently, plasticized polyvinyl chloride (PVC) (1926), low-density polyethylene (LDPE) (1933), acrylic polymer (1936), Nylone (1939), and polyethylene terephthalate (PET) (1941) were also invented and marketed [47].

MNPLs are tiny pieces of synthetic polymers (nylon, polyethylene, polyester, Teflon, and epoxy) found in ambient air, sediments, biota, fresh, and seawater [2,47,48,49,50,51]. MNPLs can be defined and classified depending on (a) their source of origin and (b) their size.

(a) There are two classes of MNPLs: the primary and the secondary. The first class includes all the MNPLs that are directly produced at micro- and nanoscale for industrial purposes (such as cosmetics, textiles, and medicine) [52,53,54]. The second category includes MNPLs created when larger plastic fragments break down through various processes, including mechanical and photochemical degradation and/or microbial actions [55,56];
(b) Four groups of MNPLs can be defined starting from their sizes: macroplastics (>25 mm), mesoplastics (5–25 mm), MPs (0.001–5 mm), and NPs (1 nm–1000 nm) [57].

The environment’s most common MNPLs are composed of various types of polymers that can be produced at different rates depending on the global demand for plastic. Koelmans et al. in 2019 found that globally in freshwater and drinking water, polymers are present in the following order: polyethylene (PE), which can be of high density (HDPE) or low density (LDPE)) ≈ polypropylene (PP) > polystyrene (PS) > polyvinyl chloride (PVC) > polyethylene terephthalate (PET) [58].

About 40–60% of the synthetic polymers are constituted by styrene-butadiene rubber (SBR), which produces MNPLs known as tire-wear particles (TWP) [57]. Paint and surface coating products contain other synthetic polymers, among which are polyester (PES), alkyds, vinyl and acrylic (co)polymers, epoxy resin, and urethane resins [59]. Besides these conventional polymers, there are other types of MNPLs produced from natural bioplastics, such as poly(lactic acid) (PLA), polyhydroxyalkanoates (PHA), poly(3-hydroxybutyrate) (PHB), poly(3-hydroxyvalerate) (PHV), and poly(3-hydroxybutyrate-co-3-hydroxyvalerate) (PHBV) [60,61].

Plastic additives aim to improve the physical features of plastic products since they act as softeners, flame retardants, UV and heat stabilizers, and production chemical agents [45]. Over 400 additives are listed by the European Chemicals Agency (ECHA) [62]. Plastic additives potentially affecting human health include phthalates and bisphenols [45]. These chemicals can pass from the polymer surface to the surrounding environment. Leaching and degradation of plasticizers and polymers are complex processes influenced by environmental conditions and the chemical properties of each additive. For example, long-lasting sunlight exposure may affect the polymer structure and make additive migration more likely. This may, on the one hand, promote additives’ release into the environment and, on the other hand, allow chemicals to adsorb on the surface [45].

Existing data indicate that bisphenol (BP) A/S/F concentrations adsorbed on MNPLs are approximately 9.52–4961 ng/g [63]; however, more investigations are required due to the small sample size. Moreover, hydrophobic interaction forces are mainly involved in maintaining the adsorption of MNPLs and BPs, and hydrogen and halogen bonds promote the adsorption. The simultaneous exposure to MNPLs and BPs can result in immune responses, neurotoxicity, inhibition of reproduction, and even reduced survival in experimental organisms [63]. However, the toxicological information on the effects of co-exposure to MNPLs and BPs is still lacking. Further research should address the ecological risks of MNPLs and BPs at environmental concentrations and promote the reduction in their flow to the natural environment and the development of non-toxic alternative materials.

Di-(2-ethylhexyl) phthalate (DEHP) is a widely used plasticizer, and it is also a known environmental endocrine disruptor that causes harm to male reproduction [64]. Due to its unstable binding to plastic compounds, it is easily released into the environment [65]. The adsorption of DEHP on MPs is higher than that of other phthalate esters (PAEs), such as dibutyl phthalate (DBP), dimethyl phthalate (DMP), or diethyl phthalate (DEP) [66], and studies in mice showed more significant disruption of spermatogenesis and more serious sperm physiological alternations after DEHP-contaminated MPs exposure than DEHP alone [66]. However, whether plastic particles could enhance the toxicity of this type of co-contaminants is still unknown.

### 2.2. Detection Techniques of MNPLs

Although MNPL investigation and detection methodologies have recently been widely investigated, the development of reliable analytical methods to measure them is still lacking, especially considering NPs [67]. The primary steps typically needed to analyze MNPLs are the MNPL sampling, sampling processing, and sample detection analysis [61].

The sampling process needs, firstly, the identification of a sampling site with adequate MNPL sources for research use, the evaluation of the biotic or abiotic factors potentially influencing MNPL properties, and, finally, the use of non-contaminated sampling tools [61]. The MNPL samplings can be performed in soil, water, and air. Soil is a significant sink for plastic debris through different mechanisms such as water runoff or atmospheric deposition, showing an uneven vertical distribution because of MNPL sources, soil type, land use, and climate [68]. In water and sediments, the sampling of MNPL is very complex, and possible factors potentially affecting the measurements should be considered, including MNPL density or shape and the environmental conditions such as currents, flows, wind, or water density [69]. Tools typically used to sample MNPLs are fine nests, manta trawls, AVANI trawls, or neuston nets [70,71]. The airborne MNPLs can be sampled passively using, for example, a stainless-steel funnel or actively by vacuum/sampling pumps or by post-deposition collection using brushes or spoons [72].

Due to the different MNPL sizes and compositions and the characteristics of the different matrices (soil, water, or air) in which they can be accumulated, the processing of the samples is very multifaceted. As an example, typically for soil samples, density flotation, filtration, and sieving are commonly used methodologies to process extrapolated MNPLs [73]. Differently, the flotation of MNPLs with lower density in more dense liquid [74,75], elutriation [76], and the Munich Plastic Sediment Separator [77] are some of the technologies used for sediment samples.

**Figure 1:**
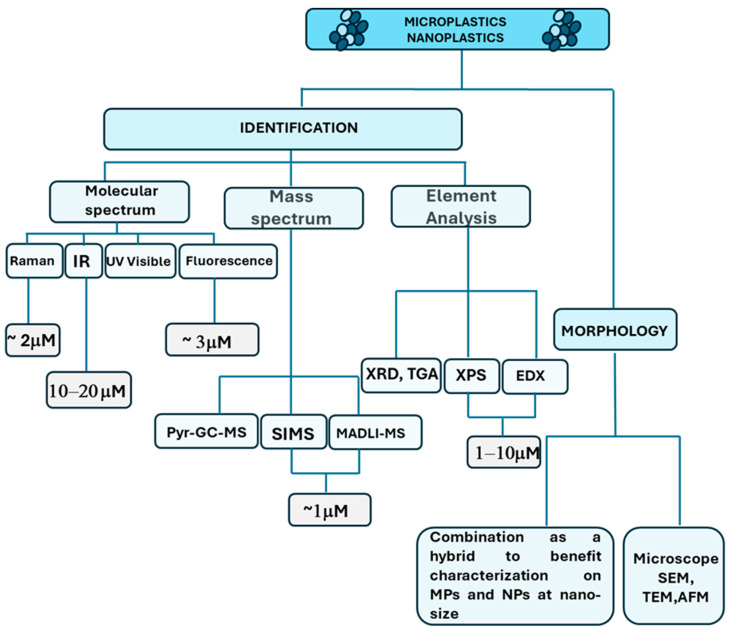
Different approaches and techniques employed for MNPL identification and characterization [78].

The detection analysis approaches can be different based on the MNPL properties. Generally, the MNPL analysis can be differentiated between physical imaging, which investigates their morphology or topology, and chemical analysis, which can provide their chemical composition. Different techniques need to be used depending on the nature of the analysis. Figure 1 summarizes the most common methods used to analyze the MNPLs.

Physical imaging includes microscopy (such as stereomicroscopy), scanning electron microscopy (SEM), transmission electron microscope (TEM), and atomic force microscopy (AFM). The microscopy techniques are usually based on fluorescence microscopy, binocular microscopy, and polarized light microscopy (PLM) [77,78,79,80]. The techniques use physical characterization (i.e., color, glossiness, cross-sectional properties, surface structure, thickness, and hardness) to identify MNPLs. SEM has always been associated with high-resolution images to obtain morphological features through surface analysis, thus uncovering the information that the particles can provide [81].

The methods used for the chemical composition of element analysis include mass spectrum and molecular spectrum. The elemental analysis consists of X-ray powder diffraction (XRD), X-ray photoelectron spectroscopy (XPS) [82], energy-dispersive X-ray spectroscopy (EDX), and thermogravimetry (TGA) [83]. Elemental analysis is often used in combination with mass spectrometry and SEM; for example, thermogravimetry coupled to mass spectrometry (TGA-MS) was developed to quantify PS-MPs in the atmosphere near an agricultural area [84]. EDX is often used in tandem with SEM [79,85]. Scanning electron microscopy–energy-dispersive X-ray spectroscopy (SEM-EDX) can analyze the morphology and elemental composition of MNPLs [79].

Fourier-transform infrared spectroscopy (FTIR) is used to identify MNPLs at the molecular level through the exposure of MNPLs to infrared radiation at different wavelengths and reference spectral libraries [86]. Micro-Fourier-transform infrared spectroscopy (μ-FTIR) and micro-attenuated total reflectance Fourier-transform infrared spectroscopy (μATR FTIR) can analyze some specific polymer types [87,88]. These represent relatively mature methods for measuring MNPLs [89]. Micro-Raman spectroscopy (μ-Raman) with Nile red can detect MNPLs with smaller size (1 μm), but the results of this analysis can be affected by additive interference [90,91]. 

Pyrolysis–gas chromatography–mass spectrometry (Pyr-GC-MS) [92] and thermal desorption–gas chromatography–mass spectrometry (TD-GC-MS) [93] can quantify the mass concentration of specific MNPLs, but the sample can be easily damaged, thus affecting the accuracy of the analysis results during the heating process [85,88]. In the latest research, a high-resolution time-of-flight aerosol mass spectrometer (HR-ToF-AMS) was used to measure PS online in laboratory-generated and environmental aerosols [94]. Although this method has a high temporal resolution, it does not allow for particle mixing, and there is some uncertainty about the elucidation of the mixture. The principles, advantages, and limitations of the currently used microscopic and analytical techniques for MNPL identification and quantification are summarized in Table 1.

## 3. Oral Exposure to MNLPs: Implications for Human Health

Although the dispersion of MNPLs in the environment is considered a global issue, knowledge concerning their impact on human health is still lacking. The literature identified three major routes for MNPL exposure: ingestion [99], inhalation [100], and dermal contacts [52]. MNPLs have been found in different foodstuff classes, such as seafood, beverages, drinking water, salt, sugar, honey, terrestrial meat, and crops and livestock [101,102]. Different classes of polymers, e.g., PP, PE, PVC, and PET, have been measured in mussels, with contamination percentages ranging between 61% and 97% of the mussels containing MNPLs and concentrations from about 0.040 ± 0.003 MPs/g wet weight (ww) in fresh mussels and up to 0.9 ± 0.1 MPs/g ww in processed mussels [89]. Polyamide (PA), PP, PVC, PS, and PET have also been identified in prawns, crabs, squid, and shrimps with MNPL diameters from 20 µm to 5000 µm [101,102]. Pyr-GC-MS detected polyvinyl chloride in oysters, prawns, squid, crabs, and sardines and a total concentration of PE between 0.04 and 2.4 mg g^−1^ of tissue [103]. Similarly, MNPLs of different chemical compositions in fish meat have been found with concentrations ranging between 0–100 µg/g and 0–2400 µg/g for PS and PE, respectively [103]. Extruded polystyrene (XPS), widely used to pack meat, contaminated chicken meat at a level ranging from 4.0 to 18.7 MP-XPS/kg of packaged meat [104]. Although the study of MNPLs in cereals, which account for 55–70% of the total diets of developing countries, is still lacking, the possible transfer of plastics to cereals could occur through MNPL presence in agricultural soils and the use of contaminated waters for irrigation or of fertilizer [101].

Human oral exposure to MNLPs represents a pressing concern in environmental health research, underscored by mounting evidence of plastic’s pervasive presence in food and beverages. Once ingested, these plastic particles encounter the gastrointestinal tract and undergo a complex array of physical and chemical transformations.

Before entering the gastrointestinal system, MNLPs first encounter saliva in the mouth. Different proteins attach to plastic particles at this level, inducing a corona formation. Dorsch et al. [105] suggested that NP size and surface chemistry dictate the interaction with human saliva proteins, which has significant implications for human plastic uptake. They used PS-MNLPs of two different sizes and three different charges, incubating them individually with saliva samples from healthy volunteers [105]. This study showed that, in saliva samples, protein profiles and relative quantities were determined by the plastic particle’s size and charge, which affect their hydrodynamic size, polydispersity, and zeta potential. These processes are dynamic and influenced by exposure times [105]. Smaller particles appeared more reactive with surrounding proteins, and when cultured with different cell lines, they affected cellular metabolic activity and genotoxicity [105].

Different studies have elucidated the ability of MNPLs to accumulate within the gastrointestinal tract, with some particles translocating across intestinal epithelial barriers and entering systemic circulation [106]. This phenomenon raises profound concerns regarding their potential to induce adverse health effects, including inflammation, oxidative stress, and dysregulation of gut microbiota composition, all of which could have significant implications for overall health and well-being [107]. The persistent nature of MNPLs exacerbates these concerns, as their prolonged permanence time in the gastrointestinal tract increases the likelihood of interactions with intestinal cells and absorption into the systemic circulation, amplifying the potential health risks for human health.

Furthermore, the physicochemical properties of MNPLs influence their capacity to adsorb and transport a wide array of organic pollutants, heavy metals, and microbial pathogens, thus serving as vectors for environmental contaminants within the human body [108]. This characteristic adsorption capability facilitates the binding of toxic chemicals onto plastic surfaces, consequently enhancing their bioavailability and prolonging their persistence in biological tissues and potentially exacerbating their toxic effects [109]. Moreover, the diminutive size of NPs raises additional concerns regarding their ability to traverse biological barriers, including cellular membranes and the blood–brain barrier, and to engage with subcellular structures such as organelles and DNA [106,110]. This enhanced capability for cellular and subcellular interaction amplifies the potential adverse effects of NPs, as it augments their likelihood of inducing cellular dysfunction, genetic mutations, and cytotoxicity within biological systems [106]. Various studies have reported MNPL accumulation in lysosomes, affecting lysosomal-associated cellular activities following cellular internalization [111,112]. The subcellular localization of NPs in mitochondria and their effects on mitochondrial function were also described [113]. However, MPs have not yet been reported to accumulate in mitochondria, but their interaction was described [114]. Altogether, data from the literature suggest that MNPLs target mitochondria, affecting all biochemical energy pathways of the cell [106]. MNPLs were also reported to damage DNA inside the nucleus [115] Finally, the abundance of ribosomal protection protein genes linked to the risk of antibiotic resistance gene propagation was reported [116]. In a recent review, Poma et al. [117] collected published studies on the epigenetics effects and gene expression modulation induced by MNPLs. Although these data are still scarce, it is becoming evident that MNPLs, whether acutely or chronically administered, can induce such changes in various model organisms.

The burgeoning field of research concerning the potential health impacts of oral exposure to MNPLs has seen significant advancements in recent years, offering valuable insights into the intricate mechanisms underlying their toxicity. For instance, the groundbreaking study by Schwabl et al. [118] represented a pivotal milestone in this field, as it revealed the presence of MPs in human stool samples. This finding indicated the remarkable resilience of plastic particles through the gastrointestinal tract and raised concerns about the potential long-term accumulation of plastic debris within the human body. Building upon this discovery, Garcia and colleagues [119] further elucidated the systemic distribution of ingested MPs by detecting their presence in various human tissues, including the liver, spleen, and kidneys.

As emerging evidence continues to underscore the potential health risks related to human exposure to MNPLs via the oral route, urgent action is required to address these concerns through comprehensive risk assessments and regulatory measures. The imperative for such initiatives is underscored by the need for interdisciplinary collaboration among researchers, policymakers, and stakeholders to develop robust methodologies for detecting and quantifying plastic contaminants in food and beverages. Moreover, elucidating the intricate mechanisms of plastic particle toxicity and evaluating their long-term health effects are critical components of these efforts [120]. Additionally, proactive measures aimed at curbing plastic pollution at its source are indispensable, necessitating improved waste management practices and advancing sustainable alternatives to conventional plastics. By undertaking these concerted efforts, society can take decisive steps toward safeguarding human health and the environment from the pervasive threat posed by plastic pollution [121].

### 3.1. The Role of MNPLs in Cell Membrane Disruption

The potential of MNPLs to disrupt cell membranes is an increasing concern in environmental health research, primarily due to their pervasive presence and possible harmful impacts on cellular integrity and function. These plastic particles have been detected across various environmental compartments, underscoring their extensive distribution [122]. Their interactions with cell membranes occur through both physical and chemical mechanisms, leading to significant structural and functional alterations [123]. Physically, MNPLs can adhere to the membrane surface, integrate into lipid bilayers, or induce mechanical stress that disrupts membrane integrity. NPs exhibit enhanced capabilities for penetrating membranes due to their high surface area-to-volume ratio, which may facilitate their internalization into cells [100]. This increased membrane interaction can compromise cell function and viability.

Chemically, these particles can release additives such as plasticizers and flame retardants, which can integrate into lipid bilayers and modify membrane fluidity, thereby affecting cell function [122]. Additionally, the hydrophobic nature of plastic surfaces encourages the adsorption of hydrophobic organic pollutants (HOPs) such as polycyclic aromatic hydrocarbons (PAHs) and polychlorinated biphenyls (PCBs). These pollutants can accumulate on plastic particles and subsequently desorb onto cell membranes upon ingestion [124]. The adsorbed pollutants can further disrupt cell membranes by altering lipid composition, changing membrane protein conformation, and affecting ion channel activity, thus distorting membrane permeability and cellular homeostasis [125].

Kihara and colleagues [126] found mild cytotoxicity and cellular uptake of PS-NPs, with no clear trend based on NP size (20 and 200 nm) or surface charge. In contrast, they observed an NP size dependency on bilayer disruption, suggesting that membrane disruption, resulting from direct interaction with PS-NPs, has little correlation with cytotoxicity. Furthermore, they reported that the level of bilayer disruption was limited to the hydrophilic headgroup, thus suggesting that transmembrane diffusion was an unlikely pathway and that cellular uptake–endocytosis is the viable mechanism. They reported that small PS-NPs (20 nm) were rarely found near chromosomes without a nuclear membrane surrounding them; however, this was not detected for larger PS-NPs (200 nm). Their findings suggest NPs can interact with chromosomes before nuclear membrane formation. Overall, PS particles’ precoating with protein coronae have been shown to reduce cytotoxicity irrespective of the corona type [126] (Table 2).

In accordance, Milillo and colleagues [127] showed that PS-NPs (800 nm) exhibited no permeation capability across membranes. They also reported internalization by target cells (human adenocarcinoma alveolar epithelial cells A549) characterized by electron-dense particles and autophagic vacuoles in a concentration-dependent manner. At higher concentrations, NPs appear dispersed inside cellular endosomes, and cells start showing signs of toxicity [127] (Table 2).

Liu et al. recently reported a deep analysis of the cellular internalization and release of MNPLs as a key finding in predicting their cytotoxicity [128]. In basophilic leukemia RBL-2H3 cells exposed to PS particles, they found that PS particles (50 and 500 nm) interact with negatively charged cell membranes through the hydrophobic interaction and Van der Waals’ forces, while 5000 nm particles were not able to absorb on the cell membrane. PS particles, sizing 50 nm and 500 nm, were internalized by cells via both passive membrane penetration and active endocytosis via clathrin-mediated and caveolin-mediated pathways (for 50 nm particles) or micropinocytosis (for 500 nm particles). Moreover, cells were able to release the accumulated intracellular particles through energy-dependent lysosomal exocytosis and energy-independent membrane penetration [128].

The disruption of cell membranes by MNPLs was linked to several adverse effects, including cytotoxicity, genotoxicity, and inflammation. The internalization of plastic particles can trigger immune responses and inflammatory cascades, releasing proinflammatory cytokines such as interleukin-6 (IL-6) and tumor necrosis factor-alpha (TNF-α), which further exacerbate cellular damage and tissue inflammation [129] (Table 2).

Understanding the mechanisms by which MNPLs disrupt cell membranes is crucial for assessing their potential health risks. By identifying these mechanisms, researchers can develop strategies to mitigate the adverse effects of these particles on human health and the environment. Continuous investigation of their interactions with cellular structures will provide information for regulatory policies and environmental protection measures.

### 3.2. MNPL Activation of Oxidative Stress and Apoptosis Pathways

The intertwined nature of oxidative stress, apoptosis, and the toxicity of MNPLs constitutes a critical area of environmental health research, drawing considerable attention to their impact on cellular homeostasis and organismal well-being. Oxidative stress derives from an altered balance between reactive oxygen species (ROS) production and the body’s ability to counteract their harmful effects through antioxidant defenses. This imbalance is a central mechanism by which various environmental stressors, including MNPLs, induce cellular damage [130]. These plastic particles can initiate ROS production upon exposure via multiple pathways, such as mitochondrial dysfunction, NADPH oxidases’ activation, and redox-sensitive signaling cascades’ disruption [87]. The elevated ROS levels can inflict oxidative damage on critical cellular macromolecules, including lipids, proteins, and DNA, impairing cellular functions and precipitating cell death.

After the mouth, the stomach is the primary organ that MPs-contaminated food or water reach along the digestive tract. Recently, Sun et al. [131] indicated that PS-MPs (50 nm and 250 nm) at environmental-related doses significantly decreased stomach organ coefficient, inhibited gastric juice and mucus secretion, disrupted gastric barrier function, and suppressed antioxidant ability in mice. They also showed that PS-MPs inhibited cell viability, increased ROS generation, and induced apoptosis through a mitochondria-dependent pathway in human gastric epithelium cells (GES-1). Simultaneously, PS-MPs decreased mitochondrial membrane potential and ATP levels, disrupted mitochondrial kinetic homeostasis, and activated the P62/Nrf2/Keap1 pathway [131] (Table 2). Another study showed that in mice receiving drinking water containing PS-NPs for 32 consecutive weeks, plastics exposure led to a significant time- and dose-dependent increase in ROS and malonaldehyde (MDA) levels and concurrently decreased glutathione peroxidase (GSH-Px) and superoxide dismutase (SOD) contents in intestinal tissues of mice [132] (Table 2). Using PS-NPs (800 nm) on the A549 cell line, Milillo and coworkers [127] found that increased production of H_2_O_2_ downstream reduced expression of cellular antioxidant network glutathione peroxidase (*Gpx*)*1*, catalase (*CAT*), *SOD1*, and *SOD2*. Additionally, they reported that the gene expression of Heme Oxygenase 1 (*HMOX1*), whose product is a ubiquitous stress-response protein, significantly increased following PS-NP treatment. This collective analysis strongly suggests that PS-NPs induce oxidative stress [127] (Table 2).

Apoptosis, also known as programmed cell death, is a tightly regulated process that eliminates damaged or stressed cells. Exposure to MNPLs can prompt apoptotic pathways through several mechanisms, including mitochondrial dysfunction, death receptor activation, and modulation of pro- and anti-apoptotic protein expression [133]. The misregulation of apoptosis due to these particles can cause tissue damage and organ dysfunction, illustrating the complex interaction between oxidative stress and apoptotic signaling in cellular responses to environmental contaminants. Previous studies have confirmed the PS microplastics’ capacity to induce apoptosis across various tissues and organs, including reproductive [134,135], cardiovascular [136], and nervous systems [137], and in a mixed form instead of a singular mode [138]. In the A549 cell line, the expression levels of the pro-apoptotic protein Bax and the effector Caspase-3 increased after just 2 h of exposure to PS-NPs (Table 2). On the other hand, the anti-apoptotic protein Bcl-2 expression decreased at the lowest concentrations, namely 10 and 100 μg/mL, and showed a slight increase at the highest concentration, i.e., 500 μg/mL [137].

The toxicity of MNPLs is complex and involves their interactions with various cellular components and organelles, resulting in significant health implications. Examining the molecular mechanisms that underlie these plastic particles’ induction of oxidative stress and apoptosis is crucial for comprehending their toxicological impacts. This understanding will allow the development of strategies to reduce their detrimental effects on the environment and human health. Moreover, comprehensive assessments of MNPL toxicity need an integrative approach combining in vitro cell culture models, animal studies, and epidemiological research.

**Table 2:** PS particle effects on cell membrane disruption, activation of oxidative stress, and apoptosis pathways.

Particle Type	Particle Size	Experimental Models	Mechanisms of Membrane Interaction/Internalization	Findings	Ref.
**PS**	20 nm 200 nm	**In vitro model** (A549 cells incubated with 10 or 50 µg/mL of PS particles) **Lipid bilayers model** (1-palmitoyl-2-oleoyl-glycero-3-phosphocholine/cholesterol tethered lipid bilayer)	Cell adherence and uptake of PS particles and PS particles/protein corona complexes, probably due to endocytotic mechanisms PS particles interact with chromosomes, and this interaction likely took place during the prophase or anaphase mitotic stages, before the nuclear membrane formation In the lipid bilayers model, membrane disruption resulted from direct interaction with small PS particles; membrane damage was limited to the POPC headgroup	**In in vitro model, the exposure to PS particles was associated with the following:** Mild cytotoxicity and cellular uptake of PS particles, with no clear trend based on particle size or surface charge (protein corona mitigated the cytotoxicity) Increase in cells granularity Cell internalization of PS particles and their adhesion to nuclear membrane PS particles with smaller size interact more favorably with chromosomes than larger particles	[126]
**PS**	800 nm	**In vitro model** (A549 cells incubated with 10 up to 500 50 µg/mL of PS-NP)	Internalization of PS particles by A549 cells by a phagocytosis mechanism Dispersion of PS particles inside cellular endosomes, concomitantly to the start of toxicity signs	**In in vitro model, the exposure to PS particles was associated with the following:** Decrease in cell viability Increase in H_2_O_2_ generation associated with reduced expression of cellular antioxidant genes *Gpx1*, *CAT*, *SOD1*, and *SOD2*, and increased expression of *HMOX1* Induction of cellular senescence and apoptosis by modulating the expression of senescence-associated secretory phenotype genes (*Cdkn1a*, *IL1A*, *IL1B*, *IL6*, and *CXCL8*) and others involved in apoptosis modulation (*BAX*, *CASP3*, and *BCL2*)	[127]
**PS**	50 nm 500 nm 5 μm	**In vitro model** (basophilic leukemia cells, RBL-2H3, incubated with 10 up to 200 mg/L of PS particles) **Cell membranes model** (using giant unilamellar vesicles, and small unilamellar vesicles)	Negatively charged PS particles (50 and 500 nm) interact with negatively charged cell membranes through the hydrophobic interaction and Van der Waals’ forces PS 5000 nm has no adsorption on the cell membrane because its large particle size and weak Brownian motion make it difficult to diffuse to the membrane surface In RBL-2H3 cells, PS 50 nm and PS 500 nm are internalized via both passive membrane penetration and active endocytosis The endocytosis of PS 50 nm occurs through the clathrin-mediated pathway, the caveolin-mediated pathway, and macropinocytosis, but endocytosis of PS 500 nm is mainly via macropinocytosis	**In in vitro model, the exposure to PS particles was associated with the following:** The accumulation in the lysosome of the endocytosed PS 50 nm and PS 500 nm The release of PS particles from cells through both energy-dependent lysosomal exocytosis and energy-independent membrane penetration	[128]
**PS**	50 nm 250 nm	**In vivo model** (C57B/L mice administered with 0.1, 0.5, and 2.5 mg/day/mouse of PS particles, by oral gavage) **In vitro model** (normal gastric epithelial cells, GES-1, incubated with 20, 40, or 80 μg/mL of PS particles)	In GES-1 cells, more 50 nm PS particles accumulated than 250 nm particles in a dose-dependent manner	**In in vivo model, the exposure to PS particles was associated with the following:** Decrease in stomach organ coefficient, gastric juice secretion, gastric mucus secretion, and gastric tight and adherent junction expression **In in vitro model, the exposure to PS particles was associated with the following:** Decreasing cell viability Increasing of ROS generation Induction of apoptosis through mitochondria-dependent pathway Decrease in mitochondrial membrane potential, ATP level, and disruption of mitochondrial kinetic homeostasis Activation of P62/Nrf2/Keap1 pathway	[131]
**PS**	50 nm	**In vivo model** (C57B/L mice administered with 0.1, 1, and 10 mg/L of PS particles, by drinking water)	Orally ingested PS particles entered the intestinal tissues of mice and upregulated expression levels of clathrin and caveolin	**In in vivo model, the exposure to PS particles was associated with the following:** Intestinal abnormalities such as villus erosion, decreased crypt numbers, and large infiltration of inflammatory cells Decrease in Occludin protein levels but not Claudin-1 and ZO-1 levels Time- and dose-dependent increase in ROS and MDA levels and concurrently decreased GSH-Px and SOD levels Increase in the proportion of B cells in MLNs and decrease in the proportion of CD8+ T cells in IELs and LPLs Elevation of pro-inflammatory cytokines IL-6, TNF-α, and IL-1β	[132]
**PS**	50 nm 500 nm 5000 nm	**In vivo model** (C57B/L mice administered with 125, 250, and 500 mg/kg of PS particles, by single oral gavage)	PS particles moved over time and accumulated in the intestine PS particles (50 and 500 nm) were found in the spleen, kidneys, heart, liver, lungs, blood, testis and epididymis, brain, and thighbone	**In in vivo model, the exposure to PS particles was associated with the following:** Increasing ROS generation in the intestine Induction of intestinal epithelial cell apoptosis and caspase-3 activation Increasing intestinal epithelium permeability and dysregulation of expression levels of E-cadherin and tight junction proteins (Claudin-1 and ZO-1)	[133]
**PS**	0.5 μm	**In vivo model** (Wistar rats exposed to 0.015, 0.15, and 1.5 mg/die PS particles, by drinking water) **In vitro model** (granulosa cells, GC, from rat ovary and treated with 1, 5, and 25 µg/mL of PS particles)	PS particles entered the ovary and GS cells (in vivo model)	**In in vivo model, the exposure to PS particles was associated with the following:** Decreasing volume of growing follicles and downregulation of anti-Müllerian hormone level Induction of oxidative stress by increasing the level of malondialdehyde and the downregulation of antioxidant enzymes (SOD, GSH-PX, and CAT) Increasing collagen fiber and fibronectin expression in ovary tissues Induction of apoptosis in ovary GS cells by the upregulation of BAX and downregulation of BCL-2 Increasing expression of fibrosis-related proteins such as Wnt, TGF-b, b-catenin, a-SMA, and collagen in ovarian tissues **In in vitro model, the exposure to PS particles was associated with the following:** Increasing protein levels of Wnt and b-catenin in GS cells	[134]
**PS**	0.5 µm	**In vivo model** (Wistar rats exposed to 0.015, 0.15, and 1.5 mg/die PS particles, by drinking water)	Not evaluated	**In in vivo model, the exposure to PS particles was associated with the following:** Decreasing number of growing follicles Induction of oxidative stress by increasing malondialdehyde levels and downregulating antioxidant enzymes (SOD, GSH-PX, and CAT) in ovary tissues Induction of pyroptosis and apoptosis of granulosa cells mediated by NLRP3/Caspase-1 signaling pathway	[135]
**PS**	0.5 µm	**In vivo model** (Wistar rats exposed to 0.5, 5, and 50 mg/L PS particles, by drinking water)	PS particles were found in cardiomyocytes	**In in vivo model, the exposure to PS particles was associated with the following:** Induction of oxidative stress in rat heart tissues by increasing malondialdehyde levels and reducing the antioxidant enzymes expression (SOD, GSH-PX, and CAT) Increasing collagen fiber and fibronectin levels in heart tissues induction of apoptosis in heart by upregulation of BAX and downregulation of BCL-2 induction of the levels of troponin I and CK-MB in serum induction of the protein expression of fibrosis-related Wnt/b-catenin signaling pathway (Wnt, b-catenin, TGF-b, a-SMA, collagen, and fibronectin)	[136]
**PS**	100 nm	**In vitro model** (primary cells, including mouse embryonic fibroblasts, mixed neuronal cells isolated from embryonic cortex, and cortical astrocytes)	PS particles deposited and accumulated in the cell cytoplasm in a concentration-dependent manner, especially near the nucleus	**In in vitro models, the exposure to PS particles was associated with the following:** Reduced viability of mixed neuronal cells but not other cells Increased neuronal apoptosis (by inducing cleaved caspase-3 levels) and reactive astrocytosis (with the increased levels of lipocalin-2 and pro-inflammatory markers TNF-α and IL-1β)	[137]

## 4. Intestinal Effects of Oral Exposure to MNPLs

Currently, there is no legislation for MNPLs as food contaminants. Consistent data gaps concerning exposure and toxicity of such particles limit the risk assessment [139]. Information about the fate of MNPLs in the gastrointestinal tract still needs to be included. Based on in vitro and in vivo data from 2016, the European Food Safety Authority (EFSA) reviewed knowledge about the uptake along the gastrointestinal tract of MNPLs [5]. The available data on toxicokinetics are only related to absorption and distribution, while there is no information about metabolism and excretion.

Moreover, it is not known whether orally ingested MPs are degraded to NPs in the gastrointestinal tract [5]. MPs over 150 μm are not absorbed; they can bind to the intestinal mucus layer and realize direct contact with the apical part of intestinal epithelial cells, thus promoting gut inflammation and local effects on the immune system. Differently, smaller particles (<150 μm) can cross the mucus barrier. Size-dependent uptake of MNPLs may involve several mechanisms, including (i) endocytosis through enterocytes; (ii) transcytosis through microfold cells (also named M-cells), a subgroup of intestinal epithelial cells in gut lymphoid tissue; (iii) persorption (i.e., the passage through gaps formed at the villous tip due to the loss of enterocytes); and (iv) paracellular uptake [140].

Although the intestinal uptake of MPs is not very efficient [141], intestinal absorption of MPs may be associated with systemic exposure levels with a toxicological relevance. The reduced size of NPs promotes their penetration deeply into organs. Results from animal studies demonstrated that once absorbed, NPs distribute to the liver, spleen, heart, lungs, thymus, reproductive organs, kidney, and brain (i.e., they cross the blood–brain barrier) [5,142].

Moreover, airborne MPs may also impact the immune system. In particular, the smallest particles (i.e., the inhalable fraction) can be absorbed through the pulmonary epithelium [143,144], reach the systemic circulation, and exert immune effects within the so-called gut–lung axis [145]. The larger particles (the extrathoracic fraction) can be transported, at least partially, to the gastrointestinal tract by mucociliary clearance. Here, they have the same fate as ingested particles. Thus, based on their size, both ingested and inhaled plastics interact with intestinal tissues, reach the circulation, and may potentially affect the immune response.

Most publications focus on plastic particles’ environmental degradation [146,147]. However, before reaching the intestinal epithelium, MNPL passage through different gastrointestinal compartments may affect their physicochemical properties and surface parameters. Nowadays, knowledge of MNPL fate along the mammalian gastrointestinal tract is still limited. Nonetheless, one of the most relevant aspects to consider is the stability of plastic materials. Low gastric pH represents the most complex chemical condition, but specialized enzymes for plastic degradation could also contribute; however, they are lacking in the mammalian intestine [45]. This suggests that, most likely, no major degradation of plastic particles occurs during digestion. Some bacteria could contribute to plastic degradation through the action of oxygenases by adding oxygen to long carbon chains, thus destabilizing the local electric charge and provoking plastic degradation [45]. However, the distribution of these enzymes is restrained because they could also degrade bacterial molecules [148,149]. It is plausible that the most critical parameter affected by digestion is the corona formation, consisting of proteins and other surrounding molecules attached to the surface in the environment and during the first contact with digestive fluids: the digestion of protein corona composition may induce a shift towards low-molecular-weight proteins [149]. Most in vitro studies ignore the phenomenon of protein corona formation, which consists of proteins from the cell culture media and may be different from environmental and digestive fluid samples.

The uptake and transport of particles up to a maximum of 5 to 10 μm in size into intestinal cells appears plausible. However, it should be considered that the previous contact with intestinal fluids can promote particle agglomeration, thus influencing the uptake compared to single particles [45]. Intracellular uptake of larger particles seems incompatible with the size of intestinal epithelial cells of about 10 μm. According to the European Food Safety Authority (EFSA), MPs have a limited bioavailability (<0.3%), and only plastics smaller in size (<150 μm) may cross the intestinal epithelium. They can also be found in tissue but are plausibly unreactive and deposited and thus not systemically bioavailable. Only particles up to 1.5 μm in size might be distributed systemically. The highest uptake and transport were found possible for the smallest particles (with size in the submicron range). A systemic distribution of plastic particles (in the nano size range) has been proposed [150,151,152]. Cellular uptake of particles > 10 μm in size could be possible in specialized cells, such as macrophages [153,154]. A paracellular transport of particles could also occur through gut leakages in the intestinal cell monolayer. Due to this phenomenon (called persorption), bigger particles could reach portal circulation [155].

Most of the studies using in vitro models to investigate plastic particle uptake are performed with the cell line Caco-2, a widely used model for human enterocytes [156]. Caco-2 cells spontaneously differentiate, and if cultured on porous membranes, they acquire a monolayer organization suitable for transport assessments [157,158,159]. Cellular uptake by Caco-2 has been shown for fluorescent plastics from 25 to 500 nm, with the highest efficiency observed for 100 nm particles [160]. Results from other studies have indicated a small translocation of PET NPs through the intestinal barrier [161].

In vivo animal studies support a low oral bioavailability of MP particles, thus suggesting a substantial fecal excretion estimated to be more than 90% through feces (depending on different features, including size, shape, and chemical composition) [5,106]. Recently, MPs have been found in human feces [118]. At least nine different types of plastics, ranging from 50 to 500 μm, mainly PP and PET, have been detected. It is noteworthy that plastic particle detection in feces indicates their oral uptake, but it cannot sustain conclusions about oral bioavailability.

### 4.1. Impact of MNPLs on Healthy Intestine

The intestinal effects of MNPLs have emerged as a complex and multifaceted area of concern within environmental health research, highlighting the potential ramifications for gut health and, in general, human well-being. Once in the gut, these particles interact with many cellular and molecular components, creating a cascade of immunological and inflammatory responses that significantly impact intestinal homeostasis.

The intricate structure of the intestinal barrier, primarily consisting of epithelial cells held together by tight junctions, plays a crucial role in regulating the passage of nutrients and other substances while preventing the entry of harmful pathogens and toxins. However, exposure to MNPLs has been shown to compromise the integrity of this barrier. These particles disrupt tight junction proteins and increase intestinal permeability [162]. Consequently, this breach allows for the translocation of MNPLs and associated toxicants across the epithelial barrier into the systemic circulation, potentially eliciting effects beyond the gut [163].

In addition, ingesting MNPLs is associated with the induction of oxidative stress within the gastrointestinal tract. These plastic particles can act as sources of ROS or induce ROS production in surrounding cells, overwhelming antioxidant defenses and provoking oxidative damage to cellular macromolecules [99]. This oxidative stress contributes to local tissue injury and inflammation and exacerbates intestinal barrier dysfunction, perpetuating a cycle of gut dysbiosis and inflammation. Moreover, the immunomodulatory properties of MNPLs have garnered significant attention due to their capacity to modulate immune responses within the gut. These plastic particles can activate immune cells such as macrophages and dendritic cells, triggering the release of pro-inflammatory cytokines and chemokines [164].

Clinical studies designed to analyze the health risks of MNPLs are not feasible in humans due to ethical concerns. Consequently, a clear understanding of the health impact of MNPLs on humans is difficult to achieve. There needs to be more awareness of the extent of MNPL absorption and accumulation in humans and the pharmacokinetic and pharmacodynamic mechanisms associated with these processes [164].

Studies assessing the effects of MNPL exposure on the gut epithelium in vivo have been performed in invertebrates, aquatic vertebrates, and zebrafish. Evidence of MNPL intestinal toxicity in mammals is emerging [165].

Table 3 summarizes studies assessing the effects of MNPLs in murine models. The main results from these studies include the capacity of PS-MPs to decrease mucus secretion significantly and the transcript levels of genes associated with mucus secretion and ion transport in the gut [18]. Evidence about the role of PS-MP exposure on intestinal inflammation in mice is controversial: while no evidence of intestinal inflammation was found in a study in which mice were exposed to PS-MPs [66], in two other studies with polyethylene MPs, mice developed histological signs of colon and duodenum inflammation and showed higher protein amounts of the innate immune receptor toll-like receptor 4 (TLR4), the pro-inflammatory transcription factor activator protein 1 (AP-1), and interferon regulatory factor 5 (IRF5) [20]. Impairments of intestinal permeability were also observed in another study with polyethylene MPs in mice [166]. These discrepancies may be explained by differences in the mouse’s genetic background, exposure schedule, and dosage.

More recently, Huang et al. [167] performed a study in C57BL/6 mice orally administered with five different sizes of PS-NPs (20, 50, 100, 200, and 500 nm). They found that after long-term oral exposure to PS-NPs 75 mg/kg (200 nm and 500 nm), significant hepatotoxicity was induced, as revealed by increased oxidative stress, liver dysfunction, and lipid metabolism disorders. Most importantly, NPs (200 nm and 500 nm) generated local immunotoxic effects by recruiting and activating inflammatory cells, i.e., neutrophils and monocytes in the liver or intestine, potentially resulting in increased pro-inflammatory cytokine secretion and tissue damage [168]. In mice receiving drinking water containing PS-NPs (0.1, 1, and 10 mg/L) for 8 consecutive months, the levels of pro-inflammatory cytokines IL-6, TNF-α, and IL-1β were markedly elevated [133]. In another study, exposure to MNPLs decreased the levels of secretory immunoglobulin A in the intestine and the differentiation of CD4+ and CD8+ T cells in mesenteric lymph nodes.

Moreover, the impact of exposure on mammalian intestinal health is influenced by the duration and particle size rather than the concentration [168]. Hirt and Body-Malapel also reviewed the studies assessing the impact of MPs on intestinal microbiota in several contests [165]. All reports showed that MP exposure causes dysbiosis in animals. The induced variations in microflora diversity and composition are likely involved in functional immune system impairments caused by MP exposure.

The intestinal immune system continuously interacts with non-pathogenic commensal organisms and innocuous food antigens, rapidly responding to infectious threats and toxins. This capacity involves several mechanisms mediated by myeloid cells, innate lymphoid cells, and T cells. These cells are resident in the intestinal lamina propria and the draining mesenteric lymph node and are critical players of the immune system. The immunotoxicity of plastics has not been directly evaluated on the intestinal immune system. However, immune cells and also those of the intestinal immune system can be a target for plastics-induced intestinal damage, as demonstrated by studies conducted mainly in invertebrates and vertebrates, showing that the immune system is compromised by exposure to MNPLs [165].

In mice, exposure to PE-MPs affects serum levels of IL1α and granulocyte colony-stimulating factor G-CSF, decreases the regulatory T-cell count, and increases the proportion of Th17 cells in splenocytes [20]. In cross-generational studies in mice, PE exposure increased blood neutrophil counts and IgA levels in dams and altered spleen lymphocytes in both dams and offspring [169]. Altogether, these results strongly support the alteration of the immune system by plastics, highlighting the need for further immunotoxicity studies.

Although the tested exposure doses of MNPLs are generally higher than the actual dose intake by humans, the damage to the intestine is not adequately considered a disease state [22,23]. Most detected changes in intestinal functions are only at the molecular transcriptional level and are not associated with significant pathological damage in the intestine [18,170,171]. This suggests that, in healthy organisms, the intestine resists the impact of MNPLs [172,173]. However, introducing MNPLs into the intestine may alter the balance of the intestinal immune system. Consequently, non-specific immune stress affecting the integrity of the intestinal barrier may occur [174,175].

**Figure 2:**
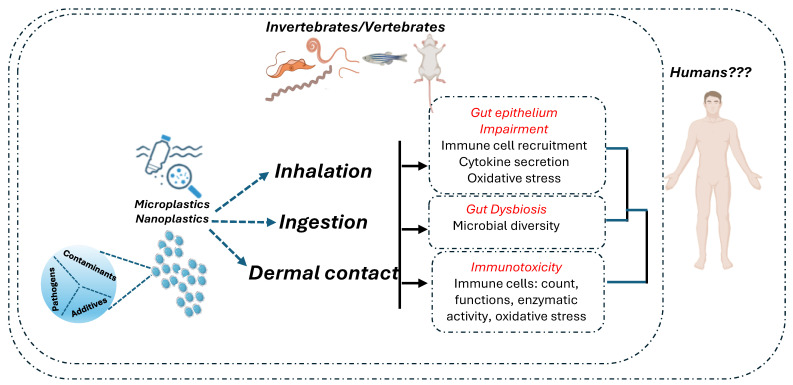
The literature identified three significant routes for MNPL exposure: ingestion, inhalation, and dermal contact. MNPL exposure leads to the impairment of oxidative and inflammatory intestinal balance and disruption of the gut’s epithelial permeability. Moreover, the effects of MNPL exposure include dysbiosis (changes in the gut microbiota) and immune cell toxicity. MNPLs contain additives and adsorb contaminants and may promote the growth of bacterial pathogens on their surfaces. Thus, they act as potential carriers of intestinal toxicants and pathogens, which can potentially increase their adverse effects. Despite the possible impact of MNPL exposure on intestinal health and immune response that has been evidenced in vertebrates and invertebrates, there is a scarcity of studies demonstrating that these effects are relevant to humans [165]. Created with biorender.com.

Studies in mice on the safety assessment of MNPLs show great particle size and dose variations, which may explain different experimental results. Therefore, clear evidence that MNPLs can cross the intestinal barrier of healthy mammals still needs to be sought [133,134,176,177]. As summarized in Table 3, in vivo studies conspicuously support the idea that MNPL exposure causes alteration of the intestinal microbiota structure of healthy individuals and induces immune stress in the intestinal mucosa. These events may induce mild intestinal inflammation at higher doses; however, severe damage to the intestine in healthy individuals has not been demonstrated [178]. Moreover, while there is evidence of the impact of MNPLs on the immune system, a large part of the studies mainly focused on the innate immune response, and the effects of MNPLs on the adaptive immune response still need to be explored. A scheme of the potential impacts of MNPL contaminations on intestinal health and immune response is reported in Figure 2.

The intestinal epithelium interacts with a wide of plastics. However, we still need more data about the amounts and features of ingested plastics. The analytical methods used to detect plastics are still limited to micro-sized particles. Moreover, these data are controversial due to the limited number of studies and poor data quality (mainly for contamination) [179]. Importantly, the lack of standardized technical methods for collection and analysis does not allow reliable interstudy. Up to now, the available data have provided only a few clues about the plastic pollutants in drinking water and in a small number of food products. Larger studies of plastics in general diets are necessary to provide a realistic estimate of oral exposure to plastics, but they still need to be performed. In this regard, it would be advisable to focus on plastics in human stools, which allow us to identify plastics with harmful effects via direct contact with the intestinal mucosa.

Although the intestinal and immunotoxic effects of NP ingestion by mammals have never been studied, it is well known that ingestion of non-plastic nanoparticles such as TiO2 nanoparticles has harmful effects, including impaired intestinal and systemic immune homeostasis and variations in the gut microbiota and metabolism [180]. This means that the presence of some NPs in the diet is likely to cause harmful effects on the intestinal and immune systems.

The toxicological effects of some types and shapes of MPs are now starting to be assessed. Moreover, the MPs most frequently studied for their in vivo effects are PS-MPs, while PP-MPs, the most abundant MPs in human feces, have been studied only to a lesser extent. The evaluation of MNPL effects on intestinal human health is further complicated by chronic exposure to MPs, which is associated with very variable effects. It is also likely that the effects of the exposure to a “mixture” of MPs (as in real life) are different from those of individual components.

**Table 3:** Effects of oral exposure to MNPLs on the healthy intestine in murine models.

Mouse Model	MNPLs	MNPLs Dose	Routes of Exposure	Time of Exposure	Key Findings	Ref.
ICR	PS 0.5 and 50 µm	100 and 1000 μg/L	Drinking water	5 weeks	Body, liver, and lipid weights (↓) Mucus secretion (↓) *Muc1* and *Klf4* mRNA levels (↓) Change of the gut microbiota composition	[173]
ICR	PS 5 µm	100 and 1000 μg/L	Drinking water	6 weeks	Mucus secretion (↓) *Muc1*, *Muc2*, *Klf4*, *Retnlb*, *Ano1*, *Cftr*, *SLC26A6*, *nkcc1,* and *Nhe3* mRNA levels (↓) Change of the gut microbiota composition Changes in serum contents of amino acids, carnitine, and succinyl acetone	[176]
C57BL/6NTac	PS 1, 4, and 10 µm	Mixture of 1 µm (1.25 mg/kg), 4 µm (25 mg/kg), and 10 µm (34 mg/kg)	Oral gavage	28 days	No effects on body and organ weights No effects on intestinal morphology No histological lesions and inflammatory response	[166]
C57BL/6	PE 10–150 μm	2–20–200 μg/g Food (6, 60, and 600 μg/d)	Food	5 weeks	In mice treated with 600 μg/day: Inflammation in colon and duodenum (↑) TLR4, AP-1, and IRF5 protein expression (↑) Change of the gut microbiota composition	[20]
CD-1	PE 45–53 μm	100 mg/kg/d	Oral gavage	30 days	Serum D-lactate levels (↑) No effect on serum diamine oxidase activity *Cyp1a2*, *Cyp1a5*, *H2BMb2*, *H2Eb1*, *Aldh8a1*, *Scarb1*, *Rdh16*, and *Gm8909* mRNA levels (↓) Change of the gut microbiota composition	[66]
ICR	PS 5 μm	100 and 1000 μg/L	Drinking water	6 weeks	*Glut2*, *Gk*, *Pk*, *Chrebp*, *Cs*, *CoA-s*, *CoA-r*, *Fabp1*, *Acox*, *PPar-α*, *Cpt1α*, *Mcad*, *Fas*, *Scd1*, *PPar-γ*, *Srebp1c*, *Acl*, *Dgat2*, *Gpat*, and *Mtp* mRNA levels (↓) Change of the gut microbiota composition *Muc1*, *Muc2*, *Muc3*, *Klf4*, *Meprin-β*, and *retnlb* mRNA levels (↓) *Nkcc1*, *Cftr*, *Slc26A3*, *Slc26A6*, *Nhe3*, and *Ano1* mRNA levels (↓) *Zo-1* and *Claudin-1* mRNA levels (↓)	[181]
ICR	PE 1–10 µm	0, 0.002, and 0.2 μg/g/d	Oral gavage	30 days	Mucus secretion (↓) *IL-8* and *IL-10* mRNA levels (↑) *IL-1β, ERK1*, and *NF-κB* mRNA levels (↓) Change of the gut microbiota composition Total protein, albumin, and globulin levels in serum (↑)	[182]
C57BL/6	PVC 2 µm	100 mg/kg	Oral gavage	60 days	Intestinal permeability (↑) Mucus secretion (↓) *Muc1, Muc2, Muc3, Klf4* mRNA levels (↓) Change of the gut microbiota composition Alterations in gut microbiome and fecal metabolic profiles	[183]
ICR	PS 0.5 μm	10 μg/mL, 50 μg/mL, and 100 μg/mL	Oral gavage	2 weeks	Thicknesses of the mucosa, muscle, flat luminal surface, crypt layer in the mid colon (↓) NLRP3, ASC, Cas-1, NF-κB, IL-6, TNF-α, and IL-1β protein expression (↑)	[184]
C57BL/6J	PS 1 µm	80 μg/kg/d	Drinking water	8 weeks	No effect on body weight and microbiome No detection in internal organs Mild pro-inflammatory profile in the colon Prolongation of viral arthritis Joint inflammation (↑)	[185]
C57BL/6J	PS 5 µm	18 and 180 μg/kg/d	Drinking water	90 days	CTX- induced liver injury (↑) Colon shortening and histological damage TNF-α, IL-6, and MDA protein expression (↑) IL-10, GSH, and SOD protein expression (↓) *Claudin-1*, *ZO-1*, *Occludin,* and *Muc-2* mRNA levels (↓) Induction of gut microbiota dysbiosis	[186]
KM	PE, PS, PP, PVC, and PET 150–300 µm	20 mg/mL	Oral gavage	1 week	Body weight in PP and PET groups (↑) Histopathological damage, decline of goblet cells, and inflammatory infiltration SOD, GSH, MDA, and POD concentrations (↑) Change of the gut microbiota composition	[187]
C57BL/6	PP 8, 70 µm	1, 10, and 100 mg/kg/d	Oral gavage	28 days	Change of colonic histopathology and ultrastructure SOD, GSH, GSH-Px, CAT, and IL-10 colonic levels (↓) Intestinal mucus secretion (↓) MDA, TNF-α, IL-1β, and IL-6 colonic levels (↑) TLR4, p50, p-p65, Bax, Caspase-9, and Caspase-3 protein expression (↑) IκBα, ZO-1, Claudin-1, Occludin, MUC1, and Bcl-2 protein expression (↓)	[188]
C57BL/6 J	PS 50, 500, and 5000 nm	2.5–500 mg/kg	Oral gavage	28 days	Intestine permeability (↑) Biodistribution in organs Mucin genes mRNA levels in the duodenum, jejunum, and ileum (↑) and in colon (↓) ROS generation Epithelial cell apoptosis *Caspase-3* mRNA level (↑)	[133]
C57BL/6	PS 200 and 800 nm	10^9^ MPLs	Oral gavage	4 weeks	Change of the gut microbiota composition Metabolic alterations in gut and brain	[189]
C57BL/6	PE 5 μm	1 and 10 mg/L	Oral administration	21 days	Liver damage Homeostasis imbalance of gut microbiota *TNF-α, IL-1β, IL-6*, and *BCL-2* mRNA levels (↓) *IL-10, BAX, BAK*, *Caspase6, Caspase7, RIPK1, RIPK3, MLKL, TLR2, IKBα, IKK, NF-κB p65*, *ASC, Caspase1*, and *NLRP3* mRNA levels (↑)	[190]
C57BL/6N	PS 100 nm	5 mg/kg bw/d	Oral gavage	28 days	Induction of liver inflammation IL-6 and TNF-α mRNA levels (↑) Change of the gut microbiota composition Changes in metabolites of the livers	[191]
KM healthy-aged	PS 1 μm	4.67 × 10^−15^, 4.67 × 10^−12^, 4.67 × 10^−9^, 4.67 × 10^−6^ mg/kg/d	Oral gavage	10 days	Aggregation of MPLs with gastrointestinal contents Liver relative weight index (↓) Liver inflammation vacuolization and structural changes SOD, CAT, 8-OHdG, IL-6, and IL-8 expression (↑) Activation of AMPK/FoxO Changes in membrane composition, energy, and amino acid metabolism	[192]
C57BL/6 J	PS 500 nm	5 mg/kg/d	Oral gavage	30 days	Intestinal damage and structural change Induction of gut microbiota dysbiosis	[132]
C57BL/6 J	PS 0.5, 5 μm	0.5 mg/mice in 100 µL sterile deionized water	Oral gavage	8 weeks	MPLs distribution in the organs TNF-α, IL-6, and IL-1β protein levels (↑) Induction of inflammation and injury in the spleen, kidney, heart, lung, and liver Intestinal barrier dysfunction in the colon Induction of gut dysbiosis Changes in intestinal metabolism	[193]
C57BL/6 J	PS 0.2, 1, 5 μm	1 mg/kg/d	Oral gavage	28 days	Colonic inflammation and oxidative stress (↑) *IL-6, IL-8, TNF-α*, and *IL-1β* mRNA levels (↑) SOD and CAT activities (↓) Mucus secretion and intestinal barrier integrity (↓) *Muc1* and *Muc2* mRNA levels (↓) Serum DAO and D-Lac concentrations (↑) ZO-1, Occludin, and Claudin-1 protein and mRNA levels (↓) Induction of intestinal barrier dysfunction through NF-κB/NLRP3/MLCK pathway	[194]
C57BL/6 J	PS 50 nm	0.1, 1, 10 mg/L	Drinking water	32 weeks	Caveolin and clathrin levels (↑) Intestinal barrier integrity (↓) Claudin-1, Occludin, and ZO-1 protein expression (↓) Induction of oxidative stress IL-1β, IL-6, and TNF-α levels (↑) Proportion of B cells in MLNs (↑) Proportion of CD8+T cells in IELs and LPLs (↓)	[132]
C57BL/6	PS 2 μm	0.5, 2 mg/kg/d	Oral gavage	8 weeks	Gut barrier dysfunction Intestinal permeability and plasma LPS levels (↑) (↑) Urinary C5a levels and C5aR1 expression (↑) Chronic kidney disease-related symptoms	[195]

Increase/exacerbation (↑); reduction (↓); polystyrene (PS); polyethylene (PE); polypropylene (PP); polyethylene terephthalate (PET); polyvinyl chloride (PVC); mononuclear leukocytes (MNLs); Intraepithelial lymphocytes (IELs); lamina propria leukocytes (LPLs).

### 4.2. MNPLs and Inflammatory Bowel Diseases

As reported above, in the intestine, the inflammatory cascade induced by MNPL exposure can disrupt mucosal immunity, alter gut microbiota composition, and impair the function of intestinal immune cells. This disruption may collectively contribute to gastrointestinal diseases, including inflammatory bowel disease (IBDs) such as Crohn’s disease (CD) and ulcerative colitis (UC) and colorectal cancer (CRC) [196].

In many countries, the number of IBD patients is increasing, reaching more than 6.8 million patients diagnosed worldwide [197,198]. IBD incidence in newly industrialized and developing countries is generally higher than in Western developed countries due to more severe environmental pollution conditions [31,32,33,34]. For this reason, IBD patients are considered high-risk individuals in the environmental impact assessment of atmospheric particulate matter [199].

**Figure 3:**
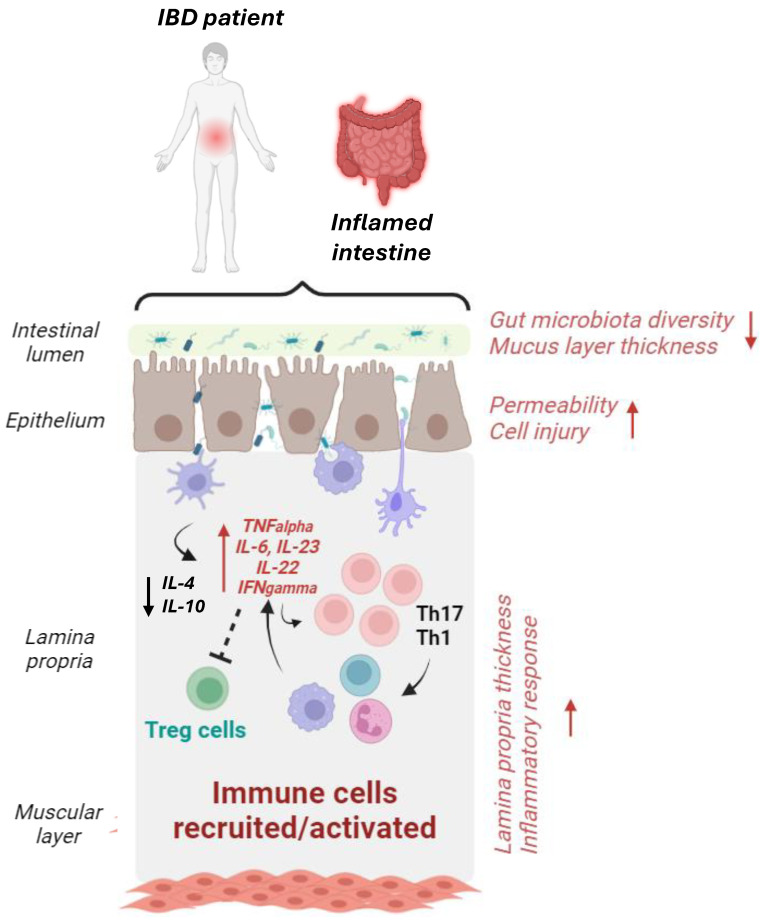
Graphical schematization of the intestinal microenvironment in IBD patients. The intestine of IBD patients is characterized by dysbiotic microbiota (i.e., lower bacterial diversity, increased pro-inflammatory bacteria, etc.), decreased mucus layer thickness, and increased epithelial permeability, which trigger immune response activation. In this setting, antigens from dysbiotic microbes activate pro-inflammatory macrophages and dendritic cells (DCs), which release pro-inflammatory cytokines (such as TNF-α and IL-6), thus stimulating Th1 and Th17 cells and the subsequent release of other pro-inflammatory mediators and the recruitment of additional immune cells. The generation of anti-inflammatory cytokines IL-10 and IL-4 from DCs and Treg cells is also reduced [24]. Increase/exacerbation (↑); reduction (↓). Created with biorender.com.

IBDs are characterized by the extensive infiltration of inflammatory cells in the intestinal mucosa, damaged structure of the intestinal barrier, and alteration of the intestinal flora, associated with diarrhea and bloody stools as the main clinical manifestations of the disease [200]. Typically, in IBD patients, a loss of the mucus layer occurs; this phenomenon promotes the attack of epithelial cells by pathogenic microorganisms [201]. Pathogenic bacteria and their product, namely lipopolysaccharide (LPS), activate immune cells, triggering an immune response in the lamina propria [202]. Activated cells, including macrophages, T lymphocytes, and B lymphocytes, secrete pro-inflammatory cytokines such as interleukin (IL)6, IL23, interferon (IFN)-γ, and tumor necrosis factor (TNF)-α, which attack intestinal epithelial cells and induce differentiation of immature immune cells in T helper 1 and 17 (Th1 and Th17) cells. Th17 cells also release pro-inflammatory mediators and recruit neutrophils [203], thus contributing to a vicious cycle of inflammation and cell damage, translating into the lamina propria thickening [202]. The regulatory T cells (Treg cells), a subgroup of immunosuppressive CD4+ T cells, produce anti-inflammatory cytokine such as IL10 and IL4 [204], and they are reduced in IBD patients (Figure 3).

Moreover, in IBDs, integrity loss of the enterocyte villus structure, tight junction (TJ) reduction, and oxidative stress have been reported. Thus, the increased cell–cell interspace promotes the crossing of harmful substances, and gut permeability is increased [205,206]. Compared to healthy individuals, in IBD patients, generally, the intestinal microbiota composition has a reduced bacteria diversity but not a significantly different number of bacteria: The abundance of *Firmicutes* phylum, *Bacteroidetes*, *Lactobacillus*, and *Bifidobacterium* decreased, while *Actinobacillus* and *Proteobacteria* were more abundant, thus suggesting that in IBDs, a decrease in the number of beneficial bacteria occurs, and the intestinal microbiota composition may also contribute to the intestinal damage [207] (Figure 3).

Recently, a relationship between MPs in feces and IBDs was described [208]. The study showed that the MP fecal concentration in IBD patients was significantly higher (41.8 items/g dm) than in healthy individuals (28.0 items/g dm). The detected MPs included 15 types, mainly sheets and fibers [208]. Moreover, the concentration of MPs and the activity level of IBDs were positively correlated. These results suggest (i) a relationship between MP exposure and the disease process or that (ii) in IBD patients, the disease process promotes MP retention.

Knowledge about the influence of MNPLs in patients with IBDs is still limited. However, several studies support the idea that MNPL exposure could aid intestinal barrier damage in these patients, thus promoting the severe development of intestinal pathology [35,36].

Recent studies in mouse models further support these suspicions. When mice were administered 100 nm PS-NPs at 1 mg/kg, 5 mg/kg, and 25 mg/kg for 28 consecutive days, inflammation and intestinal injury induced by sodium dextran sulfate (DSS) were exacerbated [209].

The most recent studies about the toxic effect of MNPLs in different animal models of IBDs are reported in Table 4. Compared to healthy controls, mice with IBDs induced by 2,4,6-trinitrobenzene sulfonic acid (TNBS) showed an increased accumulation of PS particles in the mucus layer and ulcerated regions as well as increased particle attachment to the colon mucosa [210]. In mice with acute colitis induced by DSS, PS-MPs upregulated the expression of inflammation factors (TNF-α, IL-1β, and IFN-γ) and oxidative stress factors [36]. In mice injected with LPS to cause intestinal inflammation and orally challenged with PS-NPs, LPS-induced duodenal damage and intestinal permeability were increased as well as the ROS levels and the expression of NF-κB [211].

The plastics mainly used by researchers are above 1 μm in size. However, in healthy intestinal mucosa, cell gaps have a range of 2–6 nm; thus, micron-sized plastics hardly penetrate through the tight junctions of healthy epithelial cells. In the damaged intestinal barrier, cell-to-cell gap spaces expand due to epithelial cell atrophy, thus permitting the MPs to cross the barrier. Interestingly, it has been proposed that in the intestinal lamina propria of IBD patients, the MNPLs could be engulfed by macrophages, thus inducing their immuno-activation and promoting the recruitment of more macrophages [212]. Altogether, these results indicate that IBD patients are more susceptible to MNLPs than healthy individuals and suggest the clinical need to pay attention to the toxic effects of MNLP exposure on individuals suffering from IBDs.

Recently, the comparison of MNLP content in feces from healthy volunteers and patients with IBD revealed a positive correlation between the number of fecal MNLPs and the severity of IBDs [208].

**Table 4:** Effects of oral exposure to MNPLs on the intestine in murine models of IBDs.

Mouse Model	MNPLs	MNPLs Dose	Routes of Exposure	Time of Exposure	Key Findings	Ref.
C57BL/6 and DSS-induced colitis	PS 5 µm	100 μg/L, ~1.456 × 10^6^ MPLs/L	Drinking water	42 days	IL-1*β* and IL-6 mRNA levels in colon (↑) Goblet cell number (↓) Crypt number and depth (↑) *Lgr5*, *Bmi1*, and *Olfm4* mRNA levels (↑) *c-Myc* gene and protein expression levels (↑) Bodyweight loss, diarrhea and bloody stools, and inflammation levels (↑) Severity of colitis (↑)	[213]
C57 and DSS-induced colitis	PS 5 µm	500 μg/L	Oral gavage	28 days	Liver damage (↑) IL-1β, TNF-α, and IFN-γ levels in serum (↑) TG levels in liver (↑) Intestinal permeability damage (↑) Change of lipid metabolism	[214]
C57BL/6 J and DSS-induced colitis	PS 5 µm	7280/72,800 MPLs/day	Oral gavage	14 days	Minimal effects on the intestinal barrier and liver status (MNPLs alone) *Claudin-1*, *Occludin*, and *ZO-1* mRNA levels (↓) Mucin proteins secretion (↓) Histopathological damage, inflammation, colon permeability, and immune response (↑) Risk of secondary liver injury (↑)	[215]
Lewis rats TNBS induced acute colitis	PS 0.1, 1, and 10 µm	12.5 mg particles/kg bw	Oral administration	3 days	Accumulation of MNPLs in the mucus layer and ulcerated regions (↑) Attachment to the colon tissue (↑) Size-dependent deposition of particles	[210]
C57-BL/6 mice and DSS-induced colitis	PS 5 µm	500 μg/L	Drinking water	28 days	Intestinal damage in mice with colitis (↑) PS-MPs accumulation (↑) TNF-α, IL-1β and IFN-γ, FAS, and GPx expression levels (↑) Claudin-1 and Occludin-1 expression levels (↓) Perturbation of the colonic microbial community and metabolism (↑) Pathogenic bacteria (↑)	[36]
C57BL/6 mice and LPS-induced inflammation	PS~102 nm	5 μg/g	Intraperitoneal injection	14 days	LPS-caused duodenal damage (↑) Intestinal permeability (↑) TNF-α, IL-6, and IFN-γ expression levels (↑) ZO-1, Claudin-1, and Occludin expression levels (↓) ROS production (↑) CAT, SOD, and GSH expression levels (↓) NF-κB/NLRP3 signal pathway activation (↑)	[210]

Increase/exacerbation (↑); reduction (↓); polystyrene (PS); polyethylene (PE); polypropylene (PP); polyethylene terephthalate (PET); polyvinyl chloride (PVC); mononuclear leukocytes (MNLs); intraepithelial lymphocytes (IELs); lamina propria leukocytes (LPLs); trinitrobenzenesulfonic acid (TNBS); lipopolysaccharide (LPS).

### 4.3. MNPLs in Colorectal Cancer Development and Progression

CRC is the third most common cancer worldwide, with 1.9 million cases in 2020, and in many countries, it is increasing under 50 years of age [38,39,216]. The reasons for epidemiological change are not entirely understood [217]. Still, it may be partly the result of an environmental influence, as suggested by the increased risk of early-onset CRC in individuals born after 1960 compared to previous generations [46]. This may be explained by several factors, including the increasing trends in obesity, sedentary lifestyles, smoking, the Westernization of diets, the use of antibiotics in early life [38], and the use of contraceptive pills starting from the late 1950s [218]. However, mechanistic links between this new epidemiological trend and these factors have yet to be identified [219].

It is well recognized that early-onset CRC and, in general, sporadic colorectal cancer are likely to be associated with the interaction of microbiota with the gut mucosa [220,221]. The mucosal epithelium is protected by a mucus layer that has an increased thickness from the proximal to the distal of the healthy colon [220] to act as the first line of defense for colonocytes from luminal bacteria [221]. The colon mucus layer consists of an inner sublayer, which expands distally into an outer layer. The inner mucus layer represents a barrier separating colonic bacteria from epithelium [222], while the outer mucus layer provides a nutrient-rich habitat for some colonic bacterial species [223]. It was proposed that some bacteria in this layer are associated with an increased risk of CRC [224].

By reaching the colon with the diet, MNPLs may alter the balance between the microbiota and the mucus layer and influence the colonocytes’ exposure to potentially harmful bacteria, thus promoting the incidence of CRC. To date, studies addressing the carcinogenic potential of MNPLs mainly include in vitro models and/or short-term studies in rodents, and therefore, they do not allow clear conclusions [225,226].

Carcinogenesis can involve genotoxic or non-genotoxic mechanisms [227]. In genotoxic carcinogenesis, direct DNA damage occurs due to the action of mutagens.

The chemical nature of MNPL surfaces enables them to adsorb hydrophobic compounds (including some carcinogens), charged molecules, ions such as toxic metals, and pathogenic bacteria. Thus, the health risks associated with MNLP exposures also include the delivery of MP-associated toxic chemicals to the underlying epithelium [228]. MPs may also release carcinogenic bacterial toxins to the colonic epithelium. For example, the colonic presence of *Escherichia coli* increases the risk of CRC [229] through the expression of a genotoxin [230]. MNPLs may act as a vehicle for delivering these genotoxic bacteria to the colonic epithelium; this phenomenon seems dependent on losing an intact inner mucus layer [231]. A dysbiosis condition characterized by a dietary-related shift in the abundance of bacterial species degrading colonic mucus may increase the relative abundance of genotoxic bacterial species at the surface of the colonic epithelium. The association of these bacteria with MNPLs may promote bacterial toxin delivery to the colonic epithelium, thus supporting the hypothesis that the long-term carriage of genotoxic bacteria could guide carcinogenesis in an otherwise healthy colon [232].

Non-genotoxic carcinogenesis is a process associated with the occurrence of errors during cell division. MNPLs may contribute to this process by promoting inflammation and/or delivering estrogenic compounds activating cell proliferation signaling and non-genotoxic carcinogenesis due to their interaction with estrogen receptors expressed by some intestinal epithelial cells. This phenomenon gives meaning to the idea that dietary estrogenic compounds and estrogen mimics may act as CRC promoters [233] and that some types of plastics, such as 4-nonyl phenol, are estrogen mimics [234].

It is well recognized that intestinal macrophages are essential players in preserving mucosal homeostasis and epithelial barrier integrity. Once activated, macrophages shift towards an M1 phenotype with a pro-inflammatory phenotype, thus contributing to chronic intestinal inflammation, which is, in turn, associated with an increased risk of carcinogenesis [235].

The exposure of MNPLs to a damaged mucus layer facilitates MNPL translocation through the colonocytes to the lamina propria. Here, MNPLs may be phagocytosed by resident intestinal macrophages [166] and act as a vector to deliver particle-associated toxins and surface-associated bacteria. MNPLs do not necessarily require surface-associated toxins and bacteria to elicit inflammation, as demonstrated by the finding that macrophages ingesting pristine MPs show altered cell surface markers and cytokine gene expression [212]. It has been demonstrated that when macrophages phagocytose MNPLs, a glycolysis process occurs in these cells to provide energy [212]. This supports the idea that the phenotypic plasticity of macrophages may be affected by chronic exposure to ingested MNPLs and suggests that their role in driving inflammation-associated colorectal neoplasia has been underestimated.

**Figure 4:**
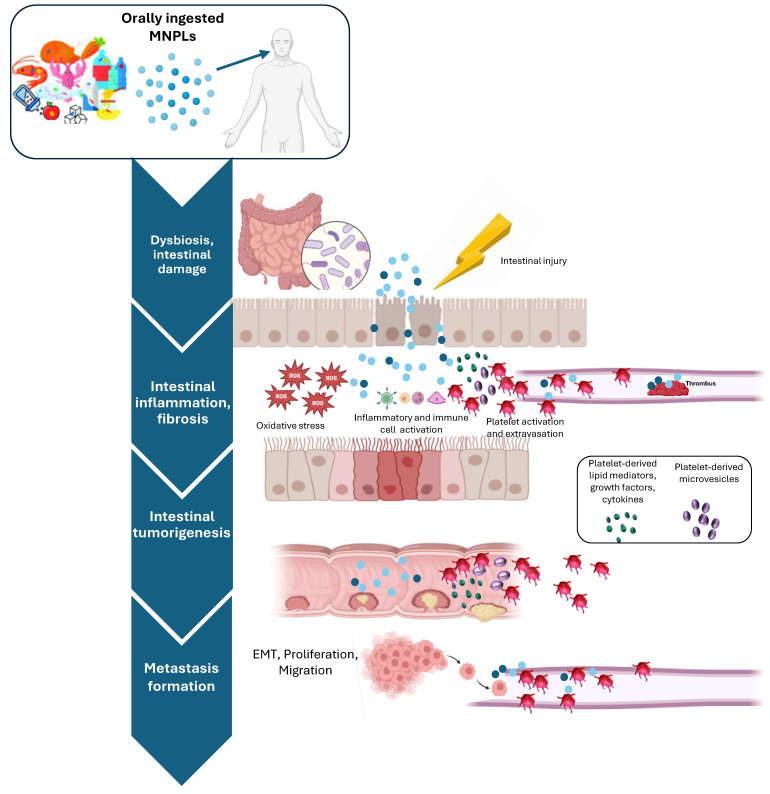
MNPLs can easily accumulate in the gastrointestinal tract. As a consequence, they can exacerbate damage to the intestinal barrier in high-risk patients (for example, with inflammatory bowel diseases), in the presence of an epithelial injury (as it occurs due to lifestyle and aging), and alter intestinal flora. These effects promote plastic particle passage through the intestinal mucus and epithelial cell layers and trigger oxidative stress, inflammatory response, impaired immune function, inhibition of cell proliferation, and tissue degeneration. Colonic mucus layer disruption may further facilitate MNPL passage in the bloodstream, thus promoting the toxic effects of MNPLs on cardiac functions, microvascular sites, and distant organs. Recently, the possible link between MNPL exposure and increased colorectal cancer (CRC) has been explored, and several potential mechanisms have been proposed. It has been suggested that platelets, beyond hemostasis and thrombosis, once activated in response to tissue damage, can mediate other processes, including immune response, inflammation, fibrosis, cancer, and metastasis formation. This is mainly due to the capacity of activated platelets to extravasate, interact, and activate other cell types such as vascular and immune cells, fibroblasts, and cancer cells through direct contact and/or the release of several soluble factors and extracellular vesicles (EVs). Created with biorender.com.

The generation of ROS species induced by MNPL exposure and the consequent oxidative stress [236] is one of the mechanisms potentially linking MNPL-induced macrophage activation and the resultant inflammation with increased risk of CRC (Figure 4) [237]. ROS generation may be promoted by the increased expression of pro-inflammatory cytokines and by their aberrant glycosylation, which contributes to mucus layer damage [237,238]. Since ROS highly reacts with other molecules, including DNA and proteins, ROS-induced oxidative damage to DNA may occur, potentially promoting oncogenic signaling and thus increasing carcinogenesis risk [239] (Figure 4).

A recently published study revealed the prolonged presence of MNPLs during cell division and provide preliminary evidence suggesting that plastic particles may promote tumor metastasis [240]. The study analyzed the interaction between PS-MNPLs (0.25 and 1 μm in size) and four human colorectal cancer cell lines, showing that the effects of the interaction depend on concentration, time, particle size, and cell type in both 2D and 3D cell cultures. In particular, they found that MNPLs exhibited high persistence in monolayer and spheroid cultures, accumulating in the non-proliferating parts of multicellular spheroids. Despite their persistence, they did not interrupt cell proliferation or division. However, particles smaller than 1 μm were able to enhance cell migration, potentially promoting metastasis. This observation aligns with recent research exploring the effect of MNPLs on breast cancer metastasis [241]. In this study, different human breast cancer cell lines (MCF-7 and MDA-MB-231) were exposed to irregularly shaped particles, reflecting the shapes most commonly present in the environment. The authors reported that plastics, when used at concentrations that were not cytotoxic (1.6 mg/mL), did not significantly affect breast cancer cells’ morphology and migration ability. However, RNA sequencing and gene expression analysis revealed that the level of cell cycle-related transcripts and genes were changed considerably in MDA-MB-231 cells exposed to MPs, thus suggesting that particles promoted metastatic capacity (Figure 4).

Altogether, these recent studies point out the persistency and bioaccumulation of MNPLs in cancer cell lines [240] and call for more studies on chronic plastic exposure and cancer progression reflecting the vast diversity of MNPLs to which humans can be exposed.

## 5. Platelet Activation in Intestinal Inflammation and Cancer: The Unexplored Contribution of MNPL Ingestion

### 5.1. Role of Platelets in Intestinal Inflammation and Cancer

Over the last decade, several pieces of evidence from pre-clinical and clinical studies corroborated the hypothesis that activated platelets participate in intestinal tumorigenesis [43,242,243,244]. Firstly, the analyses of randomized clinical trials (RCTs) revealed that the chronic use of the antiplatelet agent low-dose aspirin for cardiovascular prevention was associated with a significant reduction in the incidence and mortality of CRC but also of other types of cancer [245], thus supporting the idea that the antiplatelet effect of the drug was responsible for aspirin chemopreventive effects. In particular, it was proposed that low-dose aspirin may prevent tumorigenesis mechanisms by inhibiting platelet function [245]. From a mechanical point of view, platelets may participate in intestinal tumorigenesis by promoting intestinal inflammation and its chronic development. This may occur through interacting and activating other cells (such as immune and vascular cells, fibroblasts, and tumor cells) via direct contact and/or releasing different soluble factors and vesicles (Figure 4) [246]. In a setting of alteration of tissue microenvironment, for example, when vascular or intestinal damage occurs, platelets can be activated and in turn release several lipid mediators (such as thromboxane (TX)A_2_, prostaglandin (PG)E_2_ and 12S-Hydroxyeicosatetraenoic acid (HETE)), proteins (growth factors, proteases, and cytokines), genetic material, and extracellular vesicles (EVs), all rich in miRNAs. The release of this plethora of mediators triggers the activation of stromal cells (including fibroblasts, inflammatory cells, and endothelial cells), which, in turn, contributes to chronic inflammation and intestinal tumorigenesis [246]. Cyclooxygenase (COX)-2 expression induction, leading to the aberrant biosynthesis of pro-inflammatory and protumorigenic PGE_2_, is a key event induced by platelet activation. Using a conditional knock-out murine model with the specific deletion of COX-1 in megakaryocytes/platelets highlighted the role of platelet-derived TXA_2_ in developing intestinal inflammation and fibrosis [247]. Moreover, in Apc*^Min/+^* mice, a model of intestinal tumorigenesis, the specific deletion of COX-1 in megakaryocytes/platelets was associated with a profound reduction in the biosynthesis of platelet TXA_2_ in vivo, the decrease in the number and size of intestinal polyps, and the restraining of COX-2 expression in intestinal adenomas [248]. In inflamed intestinal tissue, platelets, being able to extravasate, can interact and activate stromal cells. In fact, in platelets co-cultured in vitro with human myofibroblasts, platelet-derived TXA_2_ promotes the induction of COX-2-dependent PGE_2_ in myofibroblasts [248].

In addition to their contribution to inflammation and the early stage of tumorigenesis, the role of platelets in cancer cell metastatization is well recognized. Platelets, by interacting with cancer cells in the bloodstream and by the release of lipid mediators (i.e., TXA_2_ and 12-HETE) and growth factors, including platelet-derived growth factor (PDGF) and transforming growth factor (TGF)β, may induce in cancer cells epithelial–mesenchymal transition (EMT) and the COX-2-signaling pathway, typically associated with a metastatic phenotype [249,250,251].

Platelets can also promote cancer development by releasing EVs, which circulate in plasma [252]. It was recently demonstrated that platelet-derived EVs were characterized by different size distribution and proteomic cargo in CRC patients compared with those from healthy individuals. Interestingly, platelet-derived EVs from CRC patients were able to promote prometastatic and prothrombotic phenotypes in cancer cells [253].

Altogether, the clinical and experimental evidence presented here suggests that low-dose aspirin, by irreversibly affecting TXA_2_ generation and inhibiting platelet activation, may lead to the drug recommendation for primary prevention of cardiovascular disease and cancer. This finding, while promising, is not without its limitations, particularly the risk of bleeding associated with the use of low-dose aspirin [254]. However, the results of ongoing and future clinical investigations, which may lead to the discovery of biomarkers for the identification of individuals with high-risk bleeding or who will benefit from low-dose aspirin treatment, could revolutionize the use of this well-known antiplatelet drug. This precision medicine strategy could significantly impact cardiovascular disease and cancer prevention, potentially changing how to approach these conditions in the future [243]. Moreover, the benefit of other antiplatelet agents, such as antagonists of the P2Y_12_ receptor for ADP and TXA_2_ receptor (TP) antagonists, should be verified by performing appropriate RCTs in these settings, further expanding our understanding and potential treatment options.

### 5.2. Effects of MNLPs on Platelet Function

The knowledge about the effects of MNPLs on platelet function is mainly derived from investigations in the cardiovascular system [109]. Due to its primary function, i.e., to drive blood circulation across the body, the cardiovascular system is highly vulnerable to MNPL toxic effects. Dysfunctional heart rates in different systems have been reported. The internalization of MNPLs by cardiac sarcomeres induced oxidative stress and subsequent apoptosis, thus probably leading to arrhythmic heart functionality [255,256,257]. Moreover, MNPLs can drive vascular toxicity through thrombosis, a considerable risk factor for cardiovascular disease.

The capacity of MNLPs to stimulate platelet aggregation is contingent upon multiple factors, including size, surface charge/modifications, and their concentration [258].

In vivo, animal studies showed that the exposure to unmodified particles did not exert effects on thrombosis, while in contrast, carboxylate–PS particles inhibited thrombus formation at 500 and 100 μg/kg, and amine–PS caused thrombosis at both 50 and 500 μg/kg [259]. Additionally, exposure to MNPLs can alter the equilibrium between pro- and anticoagulant pathways. A study conducted by Oslakovic et al. in 2012 [260] with NPs different in size and modifications explored their effects on thrombin generation and coagulation in human plasma. The aminated NPs (57 and 330 nm diameter) decreased thrombin formation in plasma, with the 57 nm group exhibiting a more substantial effect due to their binding of factors VII and IX. In contrast, carboxylated NPs acted as a surface for activation of the intrinsic pathway of blood coagulation. The ability of amine-modified NPs to bind FVII and FIX could increase the risk of bleeding, whereas the ability of carboxyl-modified NPs to activate the intrinsic pathway of coagulation could instead lead to coagulation activation and a thrombotic state [260].

In addition to the role of MNPLs on thrombin levels and blood coagulation in plasma, it was shown that MNPLs, when intravenously and intratracheally administered, lead to platelet activation and thrombus formation in animal models [259]. Some evidence proposed that P-selectin-increased expression is responsible for MNPL-dependent platelet activation [261]. Moreover, it was reported that, unlike unmodified particles, both the carboxylated and aminated NPs caused platelet aggregation by following different mechanisms [262]. Carboxylated NPs caused aggregation by classical upregulation of adhesion receptors such as glycoprotein Ⅱb/Ⅲa fibrinogen receptor (PAC-1) and P-selectin (CD62P), while aminated NPs appeared to act by perturbation of the platelet membrane via an unexplained mechanism, resulting in the display of anionic phospholipids on the external platelet plasma membrane [262]. Dobrovolskaia et al. [263] also reported that aminated NPs mediated platelet activation by inducing the perturbation of the platelet membrane through interactions with anionic phospholipids [263]. Moreover, the authors suggested that the increased platelet aggregation caused by aminated NPs could be associated with the disruption of membrane integrity and the subsequent release of mediators [263].

Most of the studies assessing the effects of MNPLs on thrombosis are performed using acute exposures at a high concentration rather than a more representative chronic exposure model. Thus, there is a need to establish more accurate animal models for a better estimation of human MNPL exposure. The chronic exposure experiments available are mainly performed in invertebrate aquatic models; however, they are less applicable to human health. Moreover, the limited knowledge in this setting is also due to the challenges associated with the analysis of MNPLs in biological samples. Recently, Wang et al. [264], by using multimodal methods, showed that MNPLs with different particle numbers, sizes, shapes, and densities accumulated in thrombi surgically collected from patients with ischemic stroke and highlighted the potential association between the mass concentration of MNPLs in thrombi and disease severity.

## 6. Conclusions and Perspectives

MNPLs are ubiquitous in the environment, and human exposure occurs from multiple sources. They may cause adverse effects in different human organ systems. Evidence shows that ingested MNPLs can easily accumulate in the gastrointestinal tract [265,266], thus causing damage to the intestinal barrier and altering intestinal flora. This promotes plastic particle passage through the intestinal mucus and epithelial cell layers and triggers oxidative stress, inflammatory response, impaired immune function, inhibition of cell proliferation, and tissue degeneration (Figure 4). Although numerous experimental studies in animal and cell culture models have evidenced the adverse biological effects of MNPLs on human health, the underlying mechanisms are still unclear.

IBDs are a group of autoimmune diseases characterized by chronic intestinal inflammation resulting from host–microbial interactions in genetically susceptible individuals. IBD patients are considered a high-risk population susceptible to the effects of MNPLs. These effects would be distinct from those on healthy people [24]. Recently, the possible link between MNPL exposure and increased CRC incidence in individuals under 50 was explored [267]. It is plausible that this changing epidemiology of CRC matches the time necessary to see the effects of the rapid increase in MNPLs in the environment [267].

Several possible mechanisms have been proposed to explain the capability of MNPLs to disrupt the colonic mucus layer, thus leading to the loss of its protective effect and a higher risk of CRC development. Recently, other functions of platelets beyond hemostasis and thrombosis have been proposed. These include immune response, inflammation, fibrosis, cancer, and metastasis formation [242]. These effects are due to the capacity of activated platelets to extravasate, interact, and activate different cell types, including vascular and immune cells, fibroblasts, and cancer cells, through direct contact and/or the release of several soluble factors and EVs [243]. It is well known that platelets can be activated in response to intestinal epithelial damage, which is possibly dependent on several risk factors, such as lifestyle and aging.

Altogether, the evidence summarized in this review supports the idea that oral MNPL exposure may potentially contribute to intestinal epithelial damage, thus promoting the chronic development of intestinal inflammation and fibrosis, mainly in high-risk populations such as IBD patients. This phenomenon may contribute to explaining the higher incidence of CRC in individuals under 50, potentially linked to the increase in human exposure to MNPLs. Finally, colonic mucus layer disruption may further facilitate MNPL passage in the bloodstream, thus promoting the toxic effects of MNPLs on cardiac functions, microvascular sites, and distant organs.

It is essential to recognize that challenges and knowledge gaps about the harmful effects of MNPL exposure on human health are still numerous. Many of the mechanisms described in MNPL toxicity have yet to be demonstrated in humans. For instance, the interaction of the mucus layer, MNPLs, and the gut microbiota requires further clarification. Moreover, most animal exposure studies are performed over short study periods, highlighting the need for longer-term studies to be designed. These challenges underscore the complexity and importance of the topic and the need for further research and understanding in this area.

It is crucial to recognize that human MNPL intake is highly variable in terms of composition and particle numbers. Geographical position, dietary intake, and lifestyle are significant factors that can influence MNPL exposure. This variability underscores the urgent need for experimental studies to clarify the distribution and accumulation of MNPLs in the human body. Limited reports have uncovered the accumulation of MNPLs in the environment or organisms, primarily due to inadequate detection technology. Therefore, it is of utmost importance that more accurate analytical methods are developed in the future to characterize MNPLs, especially for their localization in vivo. This is a pressing need in our understanding of the adverse effects of MNPLs on human health, and it requires interdisciplinary collaboration among researchers, policymakers, and stakeholders.

Moreover, proactive measures aimed at curbing plastic pollution are indispensable. These measures should be addressed to improve the technologies currently used or in development to prevent the leakage of plastic pollution or to collect existing plastic pollution. In combination with efforts to reduce the source of plastic waste and subsequent microplastic generation, policymakers should provide incentives for expanding and implementing these technologies. However, technological development cannot be separated from policy, which likewise cannot be separated from individual and industry efforts.

## Figures and Tables

**Table 1:** Analytical methods mainly used for MNPL identification and quantification.

Method	Principle	Advantages	Drawbacks	Refs.
Stereo microscopy	Samples identification directly under optical microscopes	Identification of large numbers of MPs Fast and easy Identification of shape, size, and colors	Not always applicable for particles < 500 μm Large errors due to the examiner’s subjectivity Not confirmative of the plastic nature of the particle Lack of data on transparent or small particles	[83,95]
Scanning electron microscopy (SEM)	Images are generated through the interaction of an electron beam with the sample	High-resolution image of the samples (<0.5 nm resolution)	Expensive Long time analysis Lack of information on the type of polymer Not suitable for the identification of a large number of MPs	[73,83,96]
Transmission electron Microscopy (TEM)	Sample observation (also of infinitesimal sizes) is possible due to the wave properties of the electrons, emitted by a thin filament of thermoionic material	Very high resolution (<0.1 nm) Chemical information and images of nanomaterials at a spatial resolution equal to the level of atomic dimensions are provided	Very time-consuming Not effective to visualize NPs because of their amorphous structure Sample preparation is required for particle size > 100 nm	[8,97]
Fluorescence microscopy	It collects fluorescent emission from the samples excited by a specific wavelength	Easy Useful for biological samples (cells, bacteria, etc.) and MPs Detection of transparent particles Immediate particle visualization	Laser in the ultraviolet can be harmful and toxic for the sample UV laser is phototoxic, and photodecay (bleaching of the sample) reduces the sample observation time Chemical additives can interfere with fluorescence	[8]
Fourier-transform infrared spectroscopy (FTIR)	Samples are exposed to infrared radiation, and the spectra are analyzed by comparison with known spectra in libraries	Non-destructive, fast, and reliable technique Confirmation of MP composition No false-positive or -negative data Detection of small plastic particles (<20 μm) with μ-FTIR	Not all analytes are IR-active Spectra from samples < 20 μm might not be interpretable Expensive Wavelength radiation can be a limiting detection factor Time-consuming	[8,83]
Raman apectroscopy	The excited light can be detected after laser irradiation of the sample. The frequency shift between two lights allows the identification of molecular structure and chemical components of samples.	Reliable Detection of small MPs (1 μm) and NPs (<1 μm) No false-positive or -negative data Non-destructive analysis of materials Analysis of samples in solution, gas, film, surface, solids, and single crystals is possible	Additives, color, and attached contaminants can interfere It needs sample preparation Time-consuming Expensive instrumentation Possible fragments released by adhesive polymers Interference by fluorescence induced by inorganic, organic, and (micro)biological impurities in the matrix	[8,83,98]
Pyrolysis–gas chromatography–mass spectroscopy	Thermal degradation products of MPs are identified and compared to the database for determining polymer types.	Chemical composition of samples can be identified by SEM–energy dispersive X-ray-spectroscopy (EDS) Polymer types and additives of MPs can be analyzed in one run Shape, size, and color of the samples do not affect Pyr GC-MS Reliable It is ideal for MPs in complex samples It does not require any pre-treatment of the sample Reduced time and cost	Time-consuming and destructive Morphological characterization of samples cannot be detected Lack of particle size information	[8,83]
